# The KEAP1/NRF2 axis controls LPS-induced oxidative stress, inflammasome activation and caspase-1 activity in human endothelial cells

**DOI:** 10.1371/journal.pone.0339928

**Published:** 2026-02-04

**Authors:** Alba Montero-Jodra, Maria Jesús Estebán-Amo, Silvia Patricia Fernández-Martínez, César García Martínez, Miguel Ángel de la Fuente García, Adrián García-Concejo, Marta Martín-Fernández, Eduardo Tamayo, María Simarro

**Affiliations:** 1 Department of Surgery, University of Valladolid, Valladolid, Spain; 2 Unit of Excellence, Institute of Biomedicine and Molecular Genetics (IBGM), Superior Council for Scientific Research (CSIC) – University of Valladolid (UVa), Valladolid, Spain; 3 Department of Cell Biology, Genetics, Histology and Pharmacology, University of Valladolid, Valladolid, Spain; 4 BioCritic, Group for Biomedical Research in Critical Care Medicine, Valladolid, Spain; 5 Centro de Investigación Biomédica en Red de Enfermedades Infecciosas (CIBERINFEC), Instituto de Salud Carlos III, Madrid, Spain; 6 Department of Anaesthesiology & Critical Care, Hospital Clínico Universitario de Valladolid, Valladolid, Spain; Noorda College of Osteopathic Medicine, UNITED STATES OF AMERICA

## Abstract

Endothelial cells play a critical role in the inflammatory response during sepsis, however, their metabolic adaptations to inflammatory stimuli remain much less characterized compared to immune cells. Here, we demonstrate that Human Umbilical Vein Endothelial Cells (HUVECs) do not undergo the metabolic and respiratory rewiring typically observed in macrophages following lipopolysaccharide (LPS) stimulation, a common model of inflammation during sepsis. A key metabolite in LPS-activated macrophages is itaconate, which is known for its anti-inflammatory properties. Although HUVECs do not naturally produce itaconate, we explored whether exogenous administration of the cell-permeable derivative 4-octyl itaconate (4-OI) could modulate their response to LPS. Remarkably, 4-OI treatment significantly reduced mitochondrial reactive oxygen species (mitoROS) levels in LPS-treated HUVECs, restoring them to baseline levels. This antioxidant effect was accompanied by a pronounced decrease in inflammasome activation, including suppression of ASC speck formation and caspase-1 activation. These findings suggest that 4-OI could protect endothelial cells from inflammation during sepsis in a manner similar to its role in macrophages. Mechanistically, 4-OI acts through the KEAP1/NRF2 antioxidant pathway. Silencing of KEAP1, the direct molecular target of 4-OI, resulted in a pronounced upregulation of NRF2 target genes, particularly HMOX1, with modest effects on NQO1 and no change in GCLC. NRF2 knockdown decreased HMOX1 expression and blunted 4-OI’s effects, although some residual induction persisted. Further confirming the importance of this pathway, KEAP1 silencing itself suppressed LPS-induced mitoROS, ASC speck formation, and caspase-1 activation, mimicking 4-OI treatment. Taken together, these results demonstrate that 4-OI protects endothelial cells from LPS-induced oxidative stress and inflammation primarily via the KEAP1/NRF2 axis.

## Introduction

Sepsis is a severe, life-threatening syndrome characterized by organ dysfunction resulting from a dysregulated host response to infection by bacteria, viruses, or fungi [1] and is associated with high morbidity and mortality rates [2]. Gram-negative bacteria, including *E. coli*, *Klebsiella*, *Enterobacter*, *Proteus*, and *Pseudomonas*, account for up to 40% of bacterial sepsis cases [1]. Current treatment of septic patients relies on antibiotic administration, surgical control of the infectious focus, and organ support [3]. Despite these interventions, septic shock—the most severe form of sepsis—is associated with high mortality rates (up to 40%) and remains the leading cause of death in intensive care units [4]. This underscores the urgent need for novel therapeutic approaches.

Endothelial cells (ECs), which form the inner lining of blood vessels and play a key role in vascular homeostasis, are among the first cell types to respond to circulating pathogens [5,6]. During sepsis, ECs undergo a sustained activation, acquiring a proinflammatory, procoagulant, and proadhesive phenotype, with compromised barrier function and increased apoptosis [5]. These alterations critically contribute to vascular leakage, microthrombi formation, impaired organ perfusion, and ultimately multiorgan failure [5]. In Gram-negative sepsis, ECs activation is primarily triggered by lipopolysaccharide (LPS). LPS is a major component of the outer membrane of Gram-negative bacteria and binds to the pattern recognition receptor TLR4 on ECs surfaces [7].

Recent evidence highlights a pivotal role for mitochondria in ECs dysfunction during sepsis, despite their relatively low abundance in these cells [8]. Unlike energy-demanding cells such as cardiomyocytes, ECs rely primarily on aerobic glycolysis for ATP production, suggesting that mitochondrial functions in these cells are largely regulatory [9]. In the context of sepsis, an imbalance between heightened mitochondrial reactive oxygen species (mitoROS) generation and compromised antioxidant defenses results in oxidative stress, which is a major activator of the NLRP3 inflammasome [10–13]. The NLRP3 inflammasome is a multiprotein complex that, upon activation, oligomerizes by recruiting the adaptor protein ASC and pro-caspase-1 [14]. Within this assembled complex, caspase-1 is cleaved and activated, leading to the maturation of proinflammatory cytokines IL-1β and IL-18, as well as gasdermin D-mediated pyroptosis [15]. Although targeting mitochondrial dysfunction presents a promising therapeutic strategy, investigations focused specifically on ECs remain limited.

Interestingly, itaconate, a metabolite derived from the Krebs cycle, is produced in large amounts by activated immune cells, such as LPS-treated macrophages [16]. It is synthesized from the tricarboxylic acid (TCA) cycle intermediate cis-aconitate by the enzyme cis-aconitate decarboxylase (ACOD1) [17]. Most studies investigating the role of itaconate use its derivatives, with 4-octyl itaconate (4-OI)—an esterified form of itaconate—being one of the most commonly employed due to its increased cell permeability [16]. 4-OI has been shown to efficiently reduce oxidative stress and suppress NLRP3 activation in macrophages via the KEAP1-NRF2 axis. However, its effects on ECs have barely been explored [18,19].

The aim of this study was to evaluate the potential protective effects of 4-OI in an *in vitro* sepsis model using LPS, the key activating component of Gram-negative bacteria, in primary human umbilical vein endothelial cells (HUVECs). LPS alone is a widely accepted *in vitro* model of sepsis in ECs and has been shown to increase mitoROS [20–22] and activate the NLRP3 inflammasome [23–25], ASC speck formation [26–29], caspase-1 [23–25,27,28], and IL-1β [30,31] production in various EC types. We selected LPS alone because it allows the specific assessment of early inflammasome-related inflammatory mechanisms under controlled experimental conditions, reducing the signal redundancy associated with secondary stimuli. This study is the first to show that 4-OI exerts its protective effects in LPS-treated HUVECs primarily via the KEAP1-NRF2 signaling pathway, leading to reduced oxidative stress and subsequent suppression of NLRP3 and caspase-1 activation. These findings suggest that 4-OI may serve as a promising therapeutic candidate for mitigating ECs dysfunction in Gram-negative sepsis.

## Materials and methods

### Cell line and *in vitro* sepsis model

Primary HUVECs from a single donor (ATCC, PCS-100–010) were used. HUVECs were cultured in Vascular Cell Basal Medium (ATCC, PCS-100–030) supplemented with the Endothelial Cell Growth Kit-VEGF (ATCC, PCS-100–041) to ensure optimal growth. Cells were maintained in a humidified incubator at 37°C with 5% CO_2_. In our *in vitro* sepsis model, cells were stimulated with LPS (Sigma-Aldrich, L6529) at concentrations of 1 µg/ml for either 6 hours or 24 hours to induce an inflammatory response mimicking sepsis.

### Extraction and assay of metabolites

To analyze potential alterations in the TCA metabolites of ECs, HUVECs were seeded in 15 mm diameter Petri dishes (175 cm^2^) and allowed to reach confluence before starting the experiment. Cells were treated with or without LPS (1 µg/mL) for 24 hours. After incubation, cells were harvested and metabolites were extracted [32] from the cell pellets using 1 ml of methanol/acetonitrile/water (2:2:1, v/v/v). Samples were vortexed for 30 seconds, flash-frozen in liquid nitrogen for 1 minute, and sonicated for 15 minutes. This freeze–thaw cycle was repeated once. To precipitate proteins, samples were incubated at −20°C for 1 hour. After centrifugation at 16,000 × g at 4°C for 15 minutes, the supernatant was collected in a new tube. Both the pellet and the supernatant were lyophilized. The pellet was used for protein quantification by Bradford assay. The supernatant was reconstituted in 100 µL of acetonitrile/water (1:1, v/v), sonicated for 10 minutes, centrifuged at 16,000 × g at 4°C for 15 minutes, and then stored at −80°C until sent for analysis by liquid chromatography–mass spectrometry (LC-MS). Intracellular levels of lactate, citrate, itaconate, succinate, malate, and oxaloacetate were quantified.

### Mitochondrial respiration assays

Mitochondrial function in HUVECs was assessed using the Seahorse XF Extracellular Flux Analyzer to measure the oxygen consumption rate (OCR) and the extracellular acidification rate (ECAR) using the Seahorse XF Cell Mito Stress Test kit. A total of 10,000 cells per well were seeded in Seahorse XFe24 cell culture microplates (Agilent, 100777−004). Cells were treated with or without LPS (1 µg/mL) for 24 hours. The Seahorse XF DMEM assay medium (Agilent, 103575−100) used contained 10 mM glucose (Agilent, 103577−100), 2 mM glutamine (Agilent,103579−100), and 1 mM pyruvate (Agilent, 103578−100). Three sequential injections were performed with the following reagents and concentrations: first, oligomycin (Sigma-Aldrich, 75351) (50 µg/ml); second, FCCP (Merck, C2920) (2 µM); third, a mixture of rotenone (Sigma-Aldrich, R-8875) (50 µM), antimycin A (Sigma-Aldrich, A8674) (50 µg/ml), and Hoechst 33342 (Invitrogen, H3570) (5.4 µM) for nuclear staining. Data were analyzed using the Seahorse Analytics software, generating plots for OCR, ECAR, and the various respiratory parameters.

### Silencing of NRF2 and KEAP1 using siRNAs

NRF2 and KEAP1 were silenced using Silencer Select siRNAs (Thermo Fisher Scientific, 4427037; NRF2 assay ID: s9493; KEAP1 assay ID: s18983) along with a scramble siRNA (Thermo Fisher Scientific, Control No. 1, 10025994). siRNAs were mixed at a 1:1 ratio with Lipofectamine (Invitrogen, 13778100), diluted in serum-free Opti-MEM (Life Technologies, 11058−021), and incubated for 15 minutes at room temperature prior to being added to the wells at a final concentration of 30 nM. The medium was replaced within 24 hours to prevent Lipofectamine-related toxicity. Experiments were performed 48 hours post-transfection following the protocols described above.

### Measurement of mitoROS and mitochondrial mass

To measure mitoROS, HUVECs were stained with the fluorescent probe MitoSOX Red (Life Technologies, M36008) and analyzed by flow cytometry. Cells were seeded in 6-well plates at a density of 300,000 cells per well. Cells were preincubated with or without 4-OI (50 µM) (Genochem, GWP00816083) for 1 hour, followed by the treatment with or without LPS (1 µg/mL) for an additional 24 hours. Cells were harvested and incubated with MitoSOX Red (1.75 µM) at 37°C for 15 minutes. Cells were then passed through the flow cytometer and the results were analysed using Kaluza software. To evaluate mitochondrial mass, cells were seeded and treated as described for MitoSOX Red staining. Subsequently, cells were incubated with the fluorescent probe MitoTracker Green (Invitrogen, M7514) at a final concentration of 75 nM at 37 °C for 15 minutes. After staining, cells were washed twice with PBS, fixed with 4% paraformaldehyde for 5 minutes, and then analyzed by flow cytometry. Alternatively, HUVECs were seeded on glass coverslips placed in 24-well plates at a density of 75,000 cells per well, using collagen (Sigma, C3867-1VL) at a concentration of 6 µg/cm² for coating. Cells were left untreated or treated with LPS (1 µg/ml) for 24 hours. After treatment, cells were fixed with 4% paraformaldehyde for 15 minutes. Nuclear staining was performed using Hoechst 33342 (Invitrogen, H3570) (5.4 µM), which was added simultaneously with MitoTracker Green (75 nM) for 15 minutes. Preparations were then mounted using antifade mounting media and imaged with a Leica SP5X confocal microscope. Ten images per condition were acquired at randomly selected fields using 60X magnification.

### Western Blotting

Cells were seeded in 10-mm diameter Petri dishes at a density of 2.0 × 10⁶ cells per well. Cells were preincubated with or without 4-OI (50 µM) for 1 h, followed by the treatment with or without LPS (1 µg/mL) for an additional 24 h. Proteins were extracted using RIPA buffer supplemented with protease inhibitors. A total of 50 µg of protein was loaded onto a SDS–polyacrylamide gel for separation and subsequently transferred onto a PVDF membrane (Immobilon, IPVH00005). Membranes were incubated with either mouse anti-IL-1β/IL-1F2 antibody (R&D Systems, AF-401-SP) or mouse anti- β-actin (Sigma-Aldrich, A3854) as a loading control. Detection was performed using a luminol-based chemiluminescent substrate kit (Thermo Scientific, 32106).

### Real-time PCR analysis

HUVECs were seeded in 6-well plates at a density of 300,000 cells per well. When indicated, cells were preincubated with or without 4-OI (50 µM) for 1 hour, followed by the treatment with or without LPS (1 µg/mL) for an additional 6 hours. Subsequently, total RNA was extracted using TRIzol reagent (Invitrogen,15596026). Two micrograms of RNA were used for reverse transcription with the First Strand cDNA Synthesis Kit (Molecular Biology, K1612). The resulting cDNA was used for real-time PCR using PowerUp SYBR Green Master Mix (Thermo Fisher Scientific, A25742-5ML). Specific primers were designed to amplify *HMOX1*, *NQO1*, *GCLC*, *KEAP1*, *NRF2, NLRP1, NLRP2 and NLRP3*. In all cases, *HPRT-1* was used as the housekeeping gene. The sequences of the primers are shown in S1 Table. Relative mRNA expression levels were calculated using the ΔΔCt method with HPRT-1 as the reference gene.

### Measurement of NLRP3 inflammasome activation

NLRP3 inflammasome activation was assessed using immunofluorescence by quantifying ASC speck formation, which is one of its key components. HUVECs were seeded on glass coverslips placed in 24-well plates at a density of 75,000 cells per well, using collagen at a concentration of 6 µg/cm² for coating. Cells were preincubated with or without 4-OI (50 µM) for 1 hour, followed by the treatment with or without LPS (1 µg/mL) for an additional for 24 hours. After stimulation, cells were fixed with 4% paraformaldehyde for 15 minutes and then blocked for 1 hour with a solution containing 0.1% Chicken egg albumin (ThermoScientific, 9006-59-1) and 0.3% Triton X-100 (Roche, 10789704001) in PBS 1X. Cells were then incubated overnight at 4°C with the primary anti-ASC antibody (Enzo, ADI-905–173) diluted 1:200. The next day, cells were incubated for 1 hour with Alexa Fluor 568-conjugated anti-rabbit secondary antibody (A11011, Invitrogen) diluted 1:1000, and for 15 minutes with Hoechst 33342 (5.4 µM). Samples were mounted using antifade mounting media and imaged with a Nikon 90i fluorescence microscope. Ten randomly selected fields with 20X magnification were acquired per condition. Image analysis was performed using ImageJ software.

### Analysis of caspase-1 activity

Caspase-1 activity was analyzed using a commercial kit containing the caspase-1 inhibitor reagent YVAD-FMK conjugated to FAM (BioRad, #ICT9158), a green fluorescent dye, and linked to a fluoromethyl ketone (FMK) reactive group. Active caspase-1 forms an irreversible covalent bond with the FMK group and becomes inhibited from further enzymatic activity and retain the fluorescent signal within the cell. Positive cells will retain a higher concentration of Fluorescent Labeled Inhibitors of Caspases (FLICA) and fluoresce brighter than negative cells. HUVECs were seeded in 6-well plates at a density of 300,000 cells per well. Cells were preincubated with or without 4-OI (50 µM) for 1 hour, followed by the treatment with or without LPS (1 µg/mL) for an additional for 24 hours. After treatment, cells were collected using a cell scraper and stained with the FLICA reagent (dilution 3X–6X) at 37°C for 1 hour in a CO_2_ incubator. Cells were then washed with the kit’s wash buffer, fixed, and passed through the flow cytometer and the results were analysed using Kaluza software.

### Molecular docking

Autodock vina program [33,34] was used to simulate the affinity between the proteins KEAP1, MAPK1, MAPK8 and the molecule of study 4-OI. The models used to represent the proteins were obtained from the Protein Data Bank: Keap1 = 8IXS [35], MAPK1 = 1TVO [36], MAPK8 = 4G1W [37]. The models being used were refined by eliminating any components that were not part of the protein. An exhaustiveness of 256 was established due to the small size of the molecule. The designed grid box used for each protein is provided in S2 Table. Each docking simulation was performed five times, yielding nine poses per run. From each run, only the two top-scoring poses were selected, resulting in a total of ten poses used to calculate the mean values. The ChimeraX program [38–40] was utilized to visualize and analyze the interactions between 4-OI and the proteins.

### Statistical analysis

A statistical analysis was performed using GraphPad Prism software. The data were analyzed using an unpaired, two-tailed t-test. Unless otherwise indicated, each data point in the figures represents a biological replicate. Results are expressed as the mean ± SEM. P values < 0.05 were considered statistically significant.

## Results

### LPS treatment increases non-mitochondrial respiration and mitoROS production in HUVEC cells

**Fig 1:**
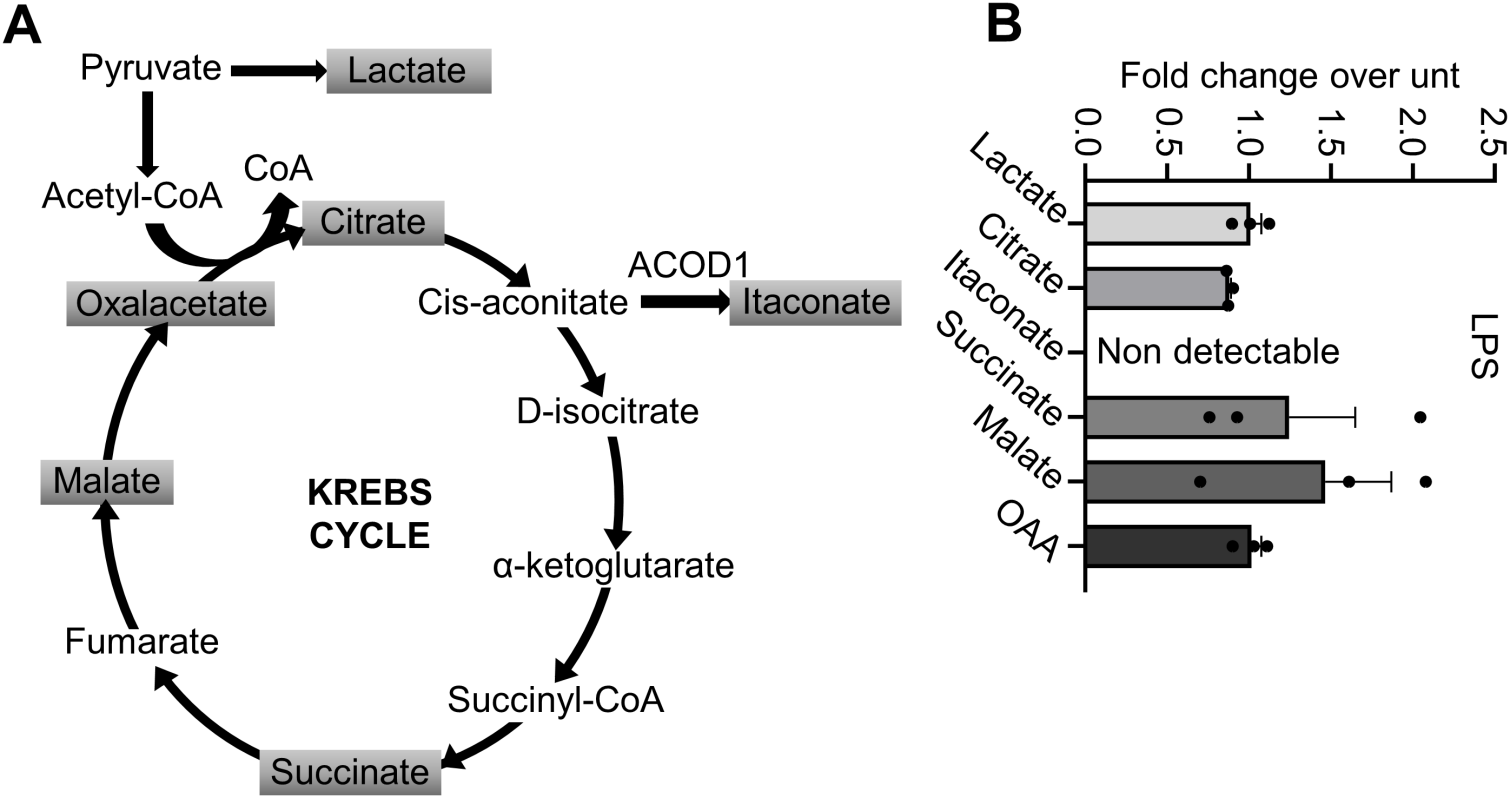
Measurement of Krebs cycle metabolites. (A) Krebs cycle representation. (B) HUVECs were treated or not with 1 µg/mL LPS for 24 hours. Then, the metabolites were extracted for UPLC/MS analysis. Each point represents a biological replicate. Data are shown as a fold change over untreated samples (unt) with the mean ± SEM.

As previously reported in immune cells such as macrophages, LPS alters the profile of TCA cycle metabolites, leading to an increase in itaconate, citrate, succinate, and lactate [41–43]. Here we investigated whether similar metabolic changes occur in HUVECs upon LPS treatment. As shown in Fig 1, LPS did not alter the levels of these metabolites in HUVECs. Notably, HUVECs did not produce itaconate upon activation. Consequently, in order to study the effects of itaconate in these cells, it must be supplemented exogenously.

**Fig 2:**
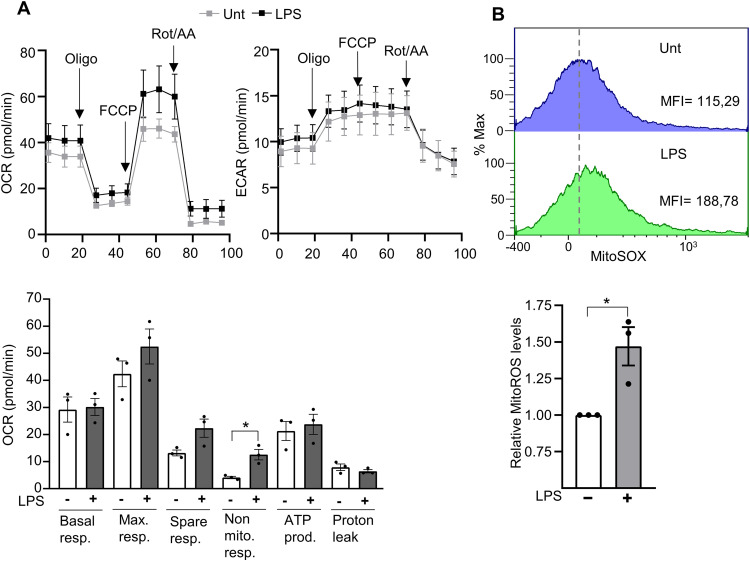
HUVEC respiration and mitoROS levels. HUVEC were treated with 1 µg/mL LPS or not for 24 hours. (A) The OCR and ECAR (top) were then measured in basal conditions and after injection of 50 µg/ml of oligomycin, 2µM of FCCP, 50µM of rotenone (rot), and 50 µg/ml of antimycin A (AA). The respiratory parameters calculated using OCR measurements are shown in the bar graph below. (B) Representative flow cytometry histogram of mitoSOX staining (top) and the graph representing the relative mitoSOX levels (bottom). Each point represents a biological replicate. MFI, mean fluorescence intensity. Data are shown as the mean ± SEM. P values meaning: *, p < 0.05; **, p < 0.01; ***, p < 0.005; ****, p < 0.001.

Next, we examined the impact of LPS on the respiratory profile of HUVECs, given that mitochondrial respiratory chain dysfunction is known to be a major source of reactive oxygen species (ROS) in cells [44]. Using the Seahorse XFp Cell Mito Stress Test, we measured both the OCR and the ECAR. As shown in Fig 2A, mitochondrial respiratory parameters, including basal respiration, maximal respiration, spare respiratory capacity, ATP production, and proton leak, did not differ significantly between LPS-treated and untreated cells. Interestingly, non-mitochondrial respiration increased significantly in HUVECs treated with LPS. In parallel, we measured mitoROS levels, which were found to be elevated in these cells as detected with the MitoSOX probe (Fig 2B).

In conclusion, although LPS does not significantly affect mitochondrial metabolite levels or key parameters of mitochondrial respiration in HUVECs, it clearly induces oxidative stress, as evidenced by increased non-mitochondrial respiration and elevated mitoROS production.

### 4-OI inhibits oxidative stress and inflammation in LPS-stimulated HUVECs

**Fig 3:**
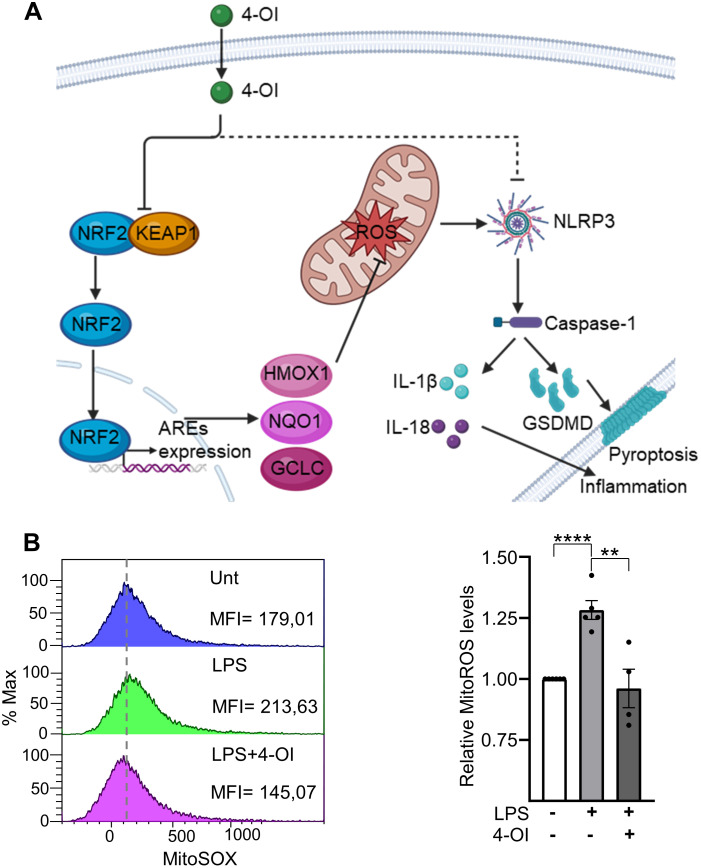
The signaling pathways of 4-OI and their impact on mitoROS levels in LPS-treated HUVECs. (A) 4-OI action in the NRF2/KEAP1 pathway. Figure was created with biorrender.com. (B) When indicated, HUVECs were pretreated with 4-OI (50 µM) for 1 hour prior to stimulation with LPS (1 µg/ml) for 24 hours. Representative flow cytometry histogram (left) of mitoSOX staining and a bar graph (right) showing the relative mitoSOX levels. Each point represents a biological replicate. MFI, mean fluorescence intensity. Data are shown as the mean ± SEM. P values meaning: *, p < 0.05; **, p < 0.01; ***, p < 0.005; ****, p < 0.001.

The anti-inflammatory and antioxidant effects of 4-OI have been extensively studied in other cell types, such as macrophages [45,46], supporting the hypothesis that it may exert similar effects in ECs. As illustrated in Fig 3A, 4-OI reduces mitoROS levels through the KEAP1/NRF2 pathway. This leads to decreased inflammasome NLRP3 activation and, consequently, decreased caspase-1 activation, which plays a role in pyroptosis and inflammation.

Next, we explored whether 4-OI could have the same effect in LPS-treated ECs. As shown in Fig 3B, 4-OI significantly decreased mitoROS levels in LPS-treated HUVECs, as measured by mitoSOX fluorescence, compared to cells treated with LPS alone. There were no significant differences between the 4-OI + LPS-treated group and the untreated cells. This indicates that 4-OI restored mitoROS levels to baseline. In parallel, mitochondrial mass was assessed using MitoTracker Green staining. As shown in S1 Fig, no significant differences were observed between conditions, indicating that the changes in mitoROS levels were not due to alterations in mitochondrial mass. Given that elevated mitoROS promotes NLRP3 inflammasome activation through oligomerization and ASC recruitment, resulting in the cleavage of procaspase-1 and production of active caspase-1, we next investigated whether 4-OI could suppress this inflammatory pathway in LPS-stimulated ECs.

The functionally relevant and best characterized inflammasome in endothelial cells is clearly NLRP3, whereas NLRP1 and NLRP2 are far less studied and their roles in ECs remain poorly defined [47,48]. We now present further evidence in support of this concept. We examined which of the three inflammasomes is upregulated by LPS to indirectly measure their functional relevance. As shown in S2 Fig, NLRP3 is the inflammasome that is most responsive to LPS stimulation and importantly, the upregulation was abolished by 4-OI.

**Fig 4:**
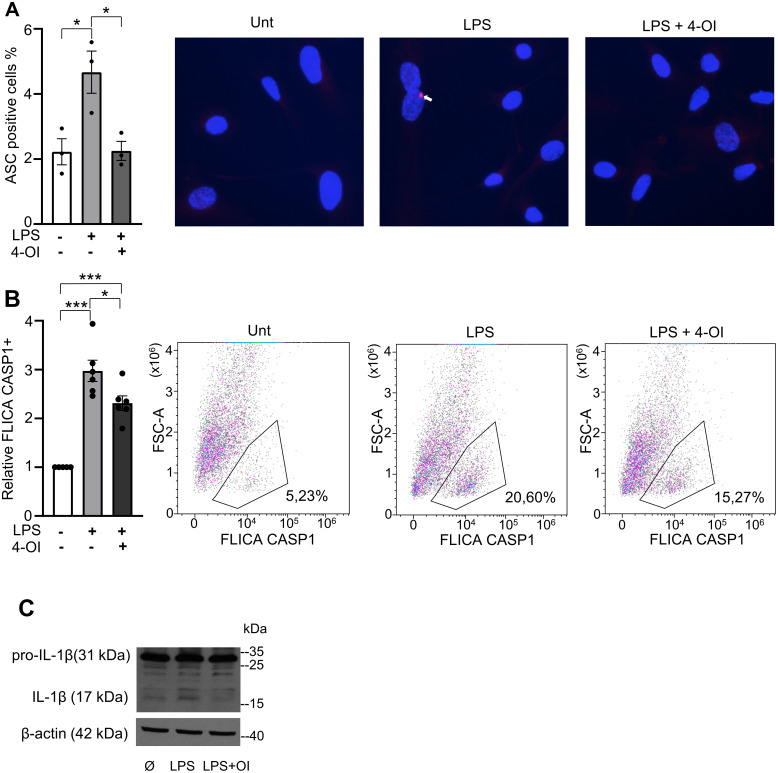
Impact of 4-OI on inflammation in LPS-treated HUVECs. When indicated, HUVECs were pretreated with 4-OI (50 µM) for 1 hour prior to stimulation with LPS (1 µg/ml) for 24 hours. (A) Bar graph (left) representig the % of ASC positive cells and representative immunofluorescence images (right) acquired at 40X magnification. ASC specks are indicated with white arrows. (B) Bar graph (left) showing the relative FLICA caspase-1 positive cells and representative flow cytometry dot plots (right) illustrating the gating strategy for caspase-1 positive cells (values are expressed in %). Each point represents a biological replicate. Data are shown as the mean ± SEM. P values meaning: *, p < 0.05; **, p < 0.01; ***, p < 0.005; ****, p < 0.001. (C) A representative Western blot of whole-cell lysates shows pro-IL-1β (31 kDa), IL-1 β (17 kDa) and β-actin as a loading control.

We found that the percentage of ASC-positive cells was significantly higher in LPS-treated HUVECs than in untreated cells. Treatment with 4-OI significantly reduced ASC speck formation in LPS-treated HUVECs, resulting in a percentage of ASC-positive cells similar to that of untreated cells (Fig 4A). To assess caspase-1 activation levels, we used a FLICA assay. As expected, relative FLICA caspase-1 levels were higher in LPS-treated HUVECs than in untreated cells. As depicted in Fig 4B, 4-OI diminished the LPS-induced increase in FLICA caspase-1 levels, although levels did not fully return to baseline. Caspase-1 processes pro–IL-1β into its 17-kDa active form, and as shown in Fig 4C, LPS triggered mature IL-1β production, and 4-OI, as expected, markedly reduced this response.

In conclusion, 4-OI restores mitoROS levels and diminishes ASC speck formation, caspase-1 activation and IL-1β maturation in LPS-treated HUVECs.

### 4-OI reduces mitochondrial ROS production by modulating the KEAP1/NRF2-mediated antioxidant response

It is known that 4-OI inhibits KEAP1 by alkylating certain cysteine residues [49,50], thereby allowing NRF2 to accumulate in the cytoplasm and translocate to the nucleus. There, it binds to antioxidant response elements (AREs) in the promoters of detoxifying and antioxidant enzymes, such as heme oxygenase 1 (HMOX1), NAD(P)H quinone oxidoreductase 1 (NQO1), and γ-glutamylcysteine ligase catalytic subunit (GCLC) (Fig 3A). This promotes the expression of these enzymes [51–54]. To explore whether 4-OI exerts its effects through this pathway in HUVECs, we performed KEAP1 and NRF2 knockdowns [55]. Silencing efficiency was verified in all experiments and reached ~80% for KEAP1 and ~70% for NRF2 by RT-PCR, as shown in S3 Fig.

**Fig 5:**
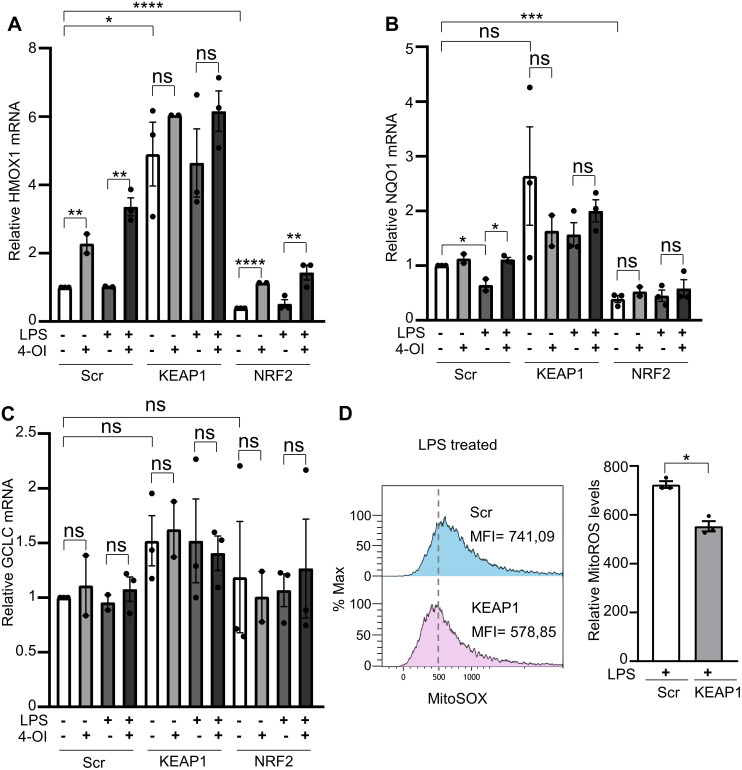
Effects of NRF2 and KEAP1 silencing on the relative expression of AREs transcripts and mitoROS levels in LPS-treated HUVECs. Following transfection with scramble, KEAP1, or NRF2 siRNA, HUVECs were pretreated with 4-OI (50 µM) for 1 hour and subsequently exposed to LPS (1 µg/ml) for 6 hours, when indicated. Bar graphs depict the relative expression levels of *HMOX1* (A), *NQO1* (B), and *GCLC* (C) transcripts relative to non-activated Scr-treated cells. (D) Representative flow cytometry histogram (left) of MitoSOX staining and a bar graph (right) showing the relative mitoSOX levels. Each point represents a biological replicate. Data are shown as the mean ± SEM. P values meaning: *, p < 0.05; **, p < 0.01; ***, p < 0.005; ****, p < 0.001; ns, non-significant.

As shown in Fig 5A, treatment with 4-OI significantly increased HMOX1 transcript levels in scramble control HUVECs, both when added alone and in combination with LPS. In contrast, treatment with LPS alone did not alter HMOX1 expression compared to untreated scramble siRNA cells. In KEAP1-silenced cells, both untreated and under all treatment conditions (4-OI, LPS, or 4-OI + LPS), HMOX1 expression was strongly elevated relative to scramble control cells, indicating a robust antioxidant protective effect. Conversely, knockdown of NRF2 resulted in a significant decrease in HMOX1 expression across all treatment conditions compared to scramble controls. 4-OI still significantly induced HMOX1 expression even in NRF2-deficient cells, both when used alone and in combination with LPS. This unexpected result is addressed in the discussion section.

As shown in Fig 5B, NQO1 transcript levels followed a similar trend to that observed for HMOX1 transcripts, with a general increase in KEAP1-silenced cells and a general decrease in NRF2-silenced cells. However, in all cases, the changes were modest (few-fold), and statistical significance was only detected in some conditions, as indicated in Fig 5B. GCLC transcript levels remained unchanged in both KEAP1- and NRF2-silenced cells (Fig 5C).

To determine whether the reduction in mitoROS observed with 4-OI was mediated through KEAP1 inactivation, we compared LPS-induced mitoROS levels in scramble control cells and KEAP1-silenced HUVECs. As shown in Fig 5D, mitoROS levels were significantly lower in LPS-treated KEAP1 knockdown cells compared to scramble controls, supporting the notion that 4-OI attenuates mitoROS production via the KEAP1/NRF2 pathway.

In conclusion, 4-OI primarily exerts its antioxidant effects through the KEAP1/NRF2 pathway, inducing key antioxidant enzymes such as HMOX1 and NQO1, which are responsible for reducing mitoROS levels. KEAP1 knockdown also mimics the protective effects of 4-OI, further supporting the central role of this signaling axis in ECs antioxidant defense.

### Silencing of KEAP-1 inhibits ASC speck formation, and caspase-1 activation in LPS-stimulated HUVECs

**Fig 6:**
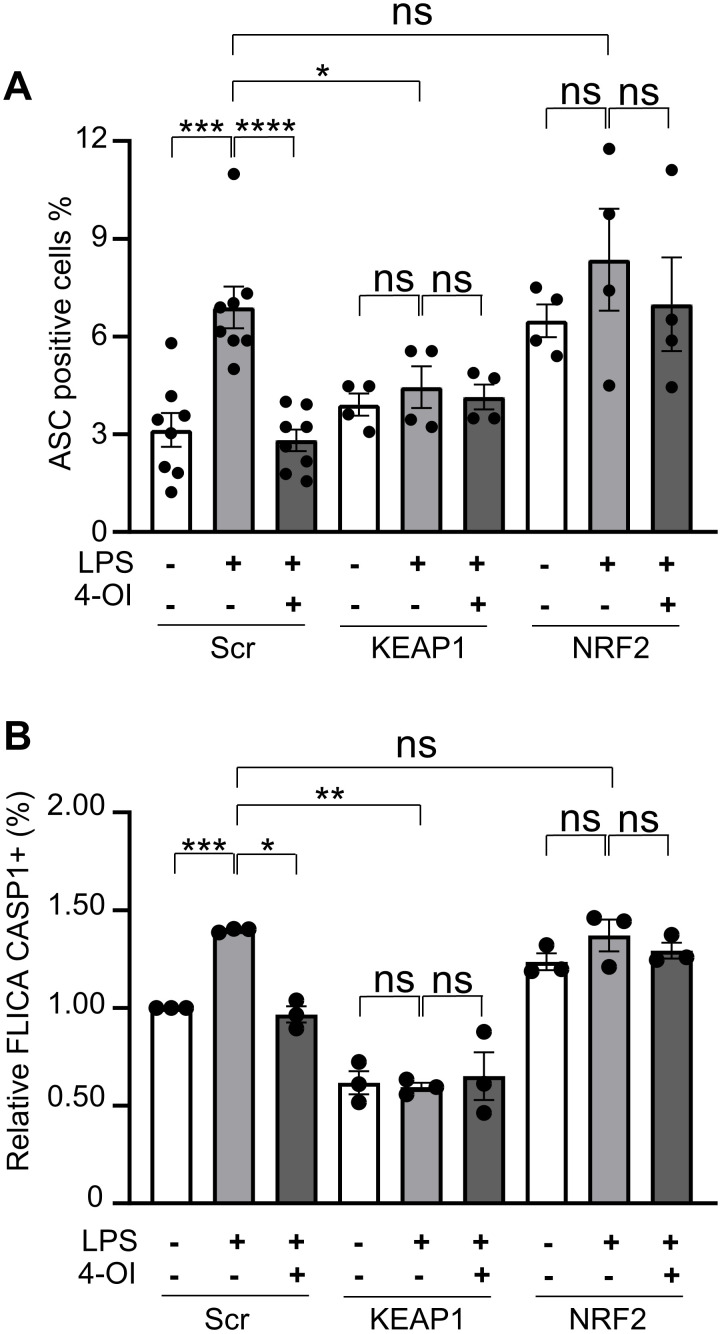
Effects of KEAP1 and NRF2 silencing on ASC specks formation and caspase-1 activation. Following transfection with scramble, KEAP1, or NRF2 siRNAs, HUVECs were pretreated with 4-OI (50 µM) for 1 hour and subsequently exposed to LPS (1 µg/ml) for 24 hours, when indicated. (A) Bar graphs representing the % of ASC positive cells and (B) bar graph showing the relative FLICA caspase-1 positive cells. Representative flow cytometry dot plots (top) illustrating the gating strategy for caspase-1 positive cells (values are expressed in %) are shown in S4 Fig. Each point represents a biological replicate. Data are shown as the mean ± SEM. P values meaning: *, p < 0.05; **, p < 0.01; ***, p < 0.005; ****, p < 0.001; ns, non-significant.

Next, we investigated whether the reduction in ASC speck formation and caspase-1 activation observed with 4-OI treatment was mediated through the KEAP1/NRF2 axis. As shown in Fig 6, 4-OI effectively prevented ASC speck formation and caspase-1 activation in LPS-treated scramble control cells. In KEAP1-silenced cells, LPS failed to induce ASC speck formation or caspase-1 activation, and these cells did not respond to 4-OI. Notably, caspase-1 activation levels in KEAP1-silenced cells were lower under all conditions compared with scramble controls. In contrast, NRF2-silenced cells exhibited elevated basal ASC speck formation and caspase-1 activation and were unresponsive to both LPS and 4-OI. Together, these findings indicate that the protective effects of 4-OI against LPS are mediated through the KEAP1/NRF2 axis (Fig 6A and 6B).

## Discussion

In this study, we demonstrate that 4-OI exerts potent antioxidant and anti-inflammatory effects in HUVECs exposed to LPS. For the first time in ECs, we show that these effects are primarily mediated via KEAP1/NRF2 signaling pathway.

While it is well established that itaconate levels increase significantly in activated immune cells such as LPS-stimulated macrophages [16], we found no such increase in LPS-treated ECs, indicating that they do not utilize itaconate as an endogenous mechanism of negative feedback regulatory mechanism. A single study [56] has quantified selected TCA cycle intermediates in LPS-stimulated ECs, reporting mild increases in aconitate (the precursor of itaconate), citrate, and α-ketoglutarate, as well as lactate, a glycolytic end-product. Meanwhile, succinate, fumarate, and malate levels remained unchanged. Under our experimental conditions, LPS-treated HUVECs showed no increase in succinate or malate. However, unlike previous findings, we did not observe increases in citrate or lactate levels. These minor discrepancies may be attributed to the heterogeneity of ECs depending on their tissue of origin [57]. The TCA cycle is functionally coupled to the mitochondrial respiration chain, and previous studies have shown that LPS can alter mitochondrial respiration in immune cells such as macrophages [58,59]. However, existing data on ECs are scarce and sometimes contradictory, while two studies report that LPS reduces maximal respiration, another study reports it increases it [60–63]. To address this gap, we assessed mitochondrial respiratory function in LPS-treated HUVECs and observed a significant increase in non-mitochondrial respiration, while the other parameters remained unchanged. The main source of non-mitochondrial (non-respiratory) oxygen consumption in endothelial cells is NADPH oxidases (NOX enzymes) – particularly NOX2 and NOX4 [64], which generate ROS. Our experiments detect an increase in mitoROS production, which is widely reported to be elevated in LPS-stimulated ECs, occurring in parallel with the rise in non-mitochondrial oxygen consumption, consistent with enhanced ROS generation from multiple sources in ECs stimulated with LPS (ECS–LPS) [22,63].

To investigate the role of itaconate in ECs, we used 4-OI, a cell-permeable derivative widely recognized as the most effective tool for studying itaconate’s biological activity. Unlike native itaconate, which cannot readily cross cell membranes, 4-OI efficiently enters the cytoplasm to exert its anti-inflammatory effects. We selected 4-OI over other derivatives such as dimethyl itaconate (DI) or 4-ethyl itaconate (4-EI) due to its lower electrophilic reactivity (reducing toxicity), its ability to be hydrolyzed intracellularly to itaconate, and its effective activation of the NRF2 pathway [16]. The anti-inflammatory and antioxidant effects of 4-OI have been well documented in macrophages, where it modifies cysteine residues on KEAP1—its direct molecular target—thereby stabilizing NRF2 and promoting the expression of cytoprotective genes [18,45,46]. In contrast, only a few studies have evaluated its protective potential in ECs [54,65–67].

Here, we show that 4-OI protects LPS-treated ECs primarily through the KEAP1/NRF2 pathway, similarly to its mechanism in macrophages. This contrasts with a recent study [65] suggesting that 4-OI acts by inhibiting MAPK/NF-κB signaling downstream of TLR4, without clarifying whether this effect is primary. In this regard, our docking studies (S5 Fig) show that 4-OI binds more strongly to KEAP1 than to the MAPK1 and MAPK8 proteins analyzed in the study by Li et al. [65], making KEAP1 the most likely primary target. Furthermore, a key strength of our study is that silencing KEAP1 abolished LPS-induced mitoROS production, ASC speck formation, and caspase-1 activation in HUVECs, underscoring the central role of the KEAP1/NRF2 pathway. This was further confirmed by NRF2 silencing, which resulted in an increased basal level of ASC speck formation and caspase-1 activation that no longer responded to the protective effect of 4-OI. Regarding the impact on the expression of antioxidant genes, both 4-OI treatment and KEAP1 knockdown strongly upregulated HMOX1 expression, whereas NRF2 silencing had the opposite effect. The residual impact of 4-OI in NRF2-deficient cells likely reflects incomplete knockdown. Interestingly, these interventions had minimal effect on NQO1 expression and did not alter GCLC transcript levels, suggesting that HMOX1 is the most functionally relevant NRF2-dependent antioxidant gene in ECs. The observation that NRF2 silencing abolishes the effects of 4-OI on ASC speck formation and caspase-1 activation, yet not on HMOX1 transcript induction, likely reflects the fact that RT-PCR–based transcript detection can capture subtle changes in HMOX1 expression that do not translate into detectable differences in downstream signaling readouts.

Additionally, the relevance of 4-OI action through KEAP1/NRF2 pathway in HUVECs has been shown in other pathological contexts. In high-glucose conditions, 4-OI reduces apoptosis and ROS production in HUVECs [54], and in a diabetic wound-healing model, 4-OI embedded in a hydrogel reduces oxidative stress and inflammation, promoting tissue regeneration via NRF2 activation [66,67].

Our findings identify the KEAP1/NRF2 axis as the principal mediator of the antioxidant and anti-inflammatory actions of 4-OI in LPS-stimulated endothelial cells, highlighting this pathway as a promising therapeutic target for developing new strategies to mitigate endothelial damage during Gram-negative sepsis.

## Supporting information

S1 Fig Evaluation of mitochondrial mass. (A) When indicated, HUVECs were preincubated for 1 hour with 4-OI (50µM) and subsequently exposed to LPS (1 µg/ml) for 24 hours. Representative flow cytometry histogram of MitoTracker Green staining (left) and a graph showing relative MitoTracker Green levels (right). Each point represents a biological replicate. Data are shown as the mean ± SEM. (B) Representative immunofluorescence confocal images of untreated and LPS-treated HUVECs stained with MitoTracker Green (60 × magnification).
(TIF)

S2 Fig Impact of 4-OI on the relative expression of NLRP1, NLRP2 and NLRP3 inflammasome transcripts in LPS-treated HUVECs. When indicated, HUVECs were pretreated with 4-OI (50 µM) for 1 hour prior to stimulation with LPS (1 µg/ml) for 6 hours. Bar graphs depict the relative expression levels of NLRP1, NLRP2 and NLRP3 transcripts relative to non-activated parental cells. Each point represents a biological replicate. Data are shown as the mean ± SEM. P values meaning: *, p < 0.05; **, p < 0.01; ***, p < 0.005; ****, p < 0.001.
(TIF)

S3 Fig siRNA efficiency validation. HUVECs were transfected with 30 nM scramble (Scr), KEAP1, or NRF2 siRNA, as indicated. Cells were collected at 48 h for transcript quantification by SYBR Green-based real-time PCR. Gene expression is expressed as fold change relative to cells treated with Scr siRNA. Each point represents a biological replicate. Data are shown as the mean ± SEM. P values meaning: *, p < 0.05; **, p < 0.01; ***, p < 0.005; ****, p < 0.001.
(TIF)

S4 Fig Effects of KEAP1 and NRF2 silencing on caspase-1 activation. Following transfection with scramble (Scr), KEAP1, or NRF2 siRNAs, HUVECs were pretreated with 4-OI (50 µM) for 1 hour and subsequently exposed to LPS (1 µg/ml) for 24 hours, where indicated. Representative flow cytometry dot plots illustrating the gating strategy for caspase-1 positive cells (values are expressed in %).
(TIF)

S5 Fig Molecular docking of 4-OI targets. 3D model illustrating the interaction between the target proteins KEAP1, MAPK1, and MAPK8 and 4-OI (light blue). The binding affinities are shown in kcal/mol. Light-blue dashed lines indicate hydrogen-bond interactions; van der Waals forces are not explicitly depicted.
(TIF)

S1 Table Primers used for real time PCR. (PDF)

S2 Table Grid-box coordinates for KEAP1, MAPK1 and MAPK8. (PDF)

S1 Dataset Source data for graphs. (XLSX)

S2 Dataset Raw microscopy data. (PDF)

S3 Dataset Raw Western blot data. (PDF)

## References

[1] Holmes CL, Anderson MT, Mobley HLT, Bachman MA. Pathogenesis of gram-negative bacteremia. Clin Microbiol Rev. 2021;34(2):e00234-20. doi: 10.1128/CMR.00234-20 33692149 PMC8549824

[2] Singer M, Deutschman CS, Seymour CW, Shankar-Hari M, Annane D, Bauer M, et al. The third international consensus definitions for sepsis and septic shock (Sepsis-3). JAMA. 2016;315(8):801–10. doi: 10.1001/jama.2016.0287 26903338 PMC4968574

[3] Zhang W, Jiang H, Wu G, Huang P, Wang H, An H, et al. The pathogenesis and potential therapeutic targets in sepsis. MedComm (2020). 2023;4(6):e418. doi: 10.1002/mco2.418 38020710 PMC10661353

[4] Black LP, Hopson C, Puskarich MA, Modave F, Booker SQ, DeVos E, et al. Racial disparities in septic shock mortality: a retrospective cohort study. Lancet Reg Health Am. 2023;29:100646. doi: 10.1016/j.lana.2023.100646 38162256 PMC10757245

[5] Joffre J, Hellman J, Ince C, Ait-Oufella H. Endothelial responses in sepsis. Am J Respir Crit Care Med. 2020;202(3):361–70. doi: 10.1164/rccm.201910-1911TR 32101446

[6] Raia L, Zafrani L. Endothelial activation and microcirculatory disorders in sepsis. Front Med (Lausanne). 2022;9:907992. doi: 10.3389/fmed.2022.907992 35721048 PMC9204048

[7] Aird WC. The role of the endothelium in severe sepsis and multiple organ dysfunction syndrome. Blood. 2003;101(10):3765–77. doi: 10.1182/blood-2002-06-1887 12543869

[8] Montero-Jodra A, de la Fuente MÁ, Gobelli D, Martín-Fernández M, Villar J, Tamayo E, et al. The mitochondrial signature of cultured endothelial cells in sepsis: Identifying potential targets for treatment. Biochim Biophys Acta Mol Basis Dis. 2024;1870(2):166946. doi: 10.1016/j.bbadis.2023.166946 37939908

[9] Caja S, Enríquez JA. Mitochondria in endothelial cells: Sensors and integrators of environmental cues. Redox Biol. 2017;12:821–7. doi: 10.1016/j.redox.2017.04.021 28448943 PMC5406579

[10] Marchi S, Guilbaud E, Tait SWG, Yamazaki T, Galluzzi L. Mitochondrial control of inflammation. Nat Rev Immunol. 2023;23(3):159–73. doi: 10.1038/s41577-022-00760-x 35879417 PMC9310369

[11] Zhou R, Yazdi AS, Menu P, Tschopp J. A role for mitochondria in NLRP3 inflammasome activation. Nature. 2011;469(7329):221–5. doi: 10.1038/nature09663 21124315

[12] Tang Y-S, Zhao Y-H, Zhong Y, Li X-Z, Pu J-X, Luo Y-C, et al. Neferine inhibits LPS-ATP-induced endothelial cell pyroptosis via regulation of ROS/NLRP3/Caspase-1 signaling pathway. Inflamm Res. 2019;68(9):727–38. doi: 10.1007/s00011-019-01256-6 31172209

[13] Xue Y, Zhang Y, Chen L, Wang Y, Lv Z, Yang L-Q, et al. Citrulline protects against LPS‑induced acute lung injury by inhibiting ROS/NLRP3‑dependent pyroptosis and apoptosis via the Nrf2 signaling pathway. Exp Ther Med. 2022;24(4):632. doi: 10.3892/etm.2022.11569 36160882 PMC9468793

[14] Nagar A, Bharadwaj R, Shaikh MOF, Roy A. What are NLRP3-ASC specks? an experimental progress of 22 years of inflammasome research. Front Immunol. 2023;14:1188864. doi: 10.3389/fimmu.2023.1188864 37564644 PMC10411722

[15] Hardy KS, Tuckey AN, Renema P, Patel M, Al-Mehdi A-B, Spadafora D, et al. ExoU induces lung endothelial cell damage and activates pro-inflammatory caspase-1 during pseudomonas aeruginosa infection. Toxins (Basel). 2022;14(2):152. doi: 10.3390/toxins14020152 35202178 PMC8878379

[16] Li Z, Zheng W, Kong W, Zeng T. Itaconate: a potent macrophage immunomodulator. Inflammation. 2023;46(4):1177–91. doi: 10.1007/s10753-023-01819-0 37142886 PMC10159227

[17] McGettrick AF, Bourner LA, Dorsey FC, O’Neill LAJ. Metabolic messengers: itaconate. Nat Metab. 2024;6(9):1661–7. doi: 10.1038/s42255-024-01092-x 39060560

[18] Hoyle C, Green JP, Allan SM, Brough D, Lemarchand E. Itaconate and fumarate derivatives inhibit priming and activation of the canonical NLRP3 inflammasome in macrophages. Immunology. 2022;165(4):460–80. doi: 10.1111/imm.13454 35137954 PMC9426622

[19] Hooftman A, Angiari S, Hester S, Corcoran SE, Runtsch MC, Ling C, et al. The immunomodulatory metabolite itaconate modifies NLRP3 and inhibits inflammasome activation. Cell Metab. 2020;32(3):468-478.e7. doi: 10.1016/j.cmet.2020.07.016 32791101 PMC7422798

[20] Wang X, Guo Y, Cui T, Zhang T, Hu W, Liu R, et al. Telomerase reverse transcriptase restores pancreatic microcirculation profiles and attenuates endothelial dysfunction by inhibiting mitochondrial superoxide production: A potential target for acute pancreatitis therapy. Biomed Pharmacother. 2023;167:115576. doi: 10.1016/j.biopha.2023.115576 37776643

[21] Ali S, Malloci M, Safiedeen Z, Soleti R, Vergori L, Vidal-Gómez X, et al. LPS-enriched small extracellular vesicles from metabolic syndrome patients trigger endothelial dysfunction by activation of TLR4. Metabolism. 2021;118:154727. doi: 10.1016/j.metabol.2021.154727 33581132

[22] Li X, Wang S, Luo M, Wang M, Wu S, Liu C, et al. Carnosol alleviates sepsis-induced pulmonary endothelial barrier dysfunction by targeting nuclear factor erythroid2-related factor 2/sirtuin-3 signaling pathway to attenuate oxidative damage. Phytother Res. 2024;38(5):2182–97. doi: 10.1002/ptr.8138 38414287

[23] Zhou X, Wu Y, Ye L, Wang Y, Zhang K, Wang L, et al. Aspirin alleviates endothelial gap junction dysfunction through inhibition of NLRP3 inflammasome activation in LPS-induced vascular injury. Acta Pharm Sin B. 2019;9(4):711–23. doi: 10.1016/j.apsb.2019.02.008 31384532 PMC6664043

[24] Zhao J, Liu Z, Chang Z. Lipopolysaccharide induces vascular endothelial cell pyroptosis via the SP1/RCN2/ROS signaling pathway. Eur J Cell Biol. 2021;100(4):151164. doi: 10.1016/j.ejcb.2021.151164 34004559

[25] Xi H, Zhang Y, Xu Y, Yang WY, Jiang X, Sha X, et al. Caspase-1 inflammasome activation mediates homocysteine-induced Pyrop-apoptosis in endothelial cells. Circ Res. 2016;118(10):1525–39. doi: 10.1161/CIRCRESAHA.116.308501 27006445 PMC4867131

[26] Liu X-H, Wu L-M, Wang J-L, Dong X-H, Zhang S-C, Li X-H, et al. Long non-coding RNA RP11-490M8.1 inhibits lipopolysaccharide-induced pyroptosis of human umbilical vein endothelial cells via the TLR4/NF-κB pathway. Immunobiology. 2021;226(5):152133. doi: 10.1016/j.imbio.2021.152133 34469785

[27] You L, Zhang D, Geng H, Sun F, Lei M. Salidroside protects endothelial cells against LPS-induced inflammatory injury by inhibiting NLRP3 and enhancing autophagy. BMC Complement Med Ther. 2021;21(1):146. doi: 10.1186/s12906-021-03307-0 34011327 PMC8136193

[28] Liu S, Zhang Y, Liang X, Yin L, He C. α-Cyperone Alleviates LPS-induced pyroptosis in rat aortic endothelial cells via the PI3K/AKT signaling pathway. Pharmaceuticals (Basel). 2025;18(3):303. doi: 10.3390/ph18030303 40143082 PMC11945463

[29] Ji J, Ye W, Sun G. lncRNA OIP5-AS1 knockdown or miR-223 overexpression can alleviate LPS-induced ALI/ARDS by interfering with miR-223/NLRP3-mediated pyroptosis. J Gene Med. 2022;24(4):e3385. doi: 10.1002/jgm.3385 34346534

[30] Li R, Fan X, He Z, Shen H, Wu H, Chen J, et al. IL-1β-stimulated bone mesenchymal stem cell-derived exosomes promote cutaneous wound healing by inhibiting SIRT6/NLRP3 pathway. Int Immunopharmacol. 2025;166:115566. doi: 10.1016/j.intimp.2025.115566 40976046

[31] Ding R, Sun X, Yi B, Liu W, Kazama K, Xu X, et al. Nur77 attenuates inflammasome activation by inhibiting caspase-1 expression in pulmonary vascular endothelial cells. Am J Respir Cell Mol Biol. 2021;65(3):288–99. doi: 10.1165/rcmb.2020-0524OC 33971110 PMC8485996

[32] Márquez S, Fernández JJ, Mancebo C, Herrero-Sánchez C, Alonso S, Sandoval TA, et al. Tricarboxylic acid cycle activity and remodeling of glycerophosphocholine lipids support cytokine induction in response to fungal patterns. Cell Rep. 2019;27(2):525-536.e4. doi: 10.1016/j.celrep.2019.03.033 30970255

[33] Eberhardt J, Santos-Martins D, Tillack AF, Forli S. AutoDock Vina 1.2.0: New docking methods, expanded force field, and python bindings. J Chem Inf Model. 2021;61(8):3891–8. doi: 10.1021/acs.jcim.1c00203 34278794 PMC10683950

[34] Trott O, Olson AJ. AutoDock Vina: improving the speed and accuracy of docking with a new scoring function, efficient optimization, and multithreading. J Comput Chem. 2010;31(2):455–61. doi: 10.1002/jcc.21334 19499576 PMC3041641

[35] Otake K, Ubukata M, Nagahashi N, Ogawa N, Hantani Y, Hantani R, et al. Methyl and fluorine effects in novel orally bioavailable keap1-Nrf2 PPI Inhibitor. ACS Med Chem Lett. 2023;14(5):658–65. doi: 10.1021/acsmedchemlett.3c00067 37197451 PMC10184158

[36] Ohori M, Kinoshita T, Okubo M, Sato K, Yamazaki A, Arakawa H, et al. Identification of a selective ERK inhibitor and structural determination of the inhibitor-ERK2 complex. Biochem Biophys Res Commun. 2005;336(1):357–63. doi: 10.1016/j.bbrc.2005.08.082 16139248

[37] Kuglstatter A, Shao A. Crystal structure of JNK1 in complex with JIP1 peptide and 7-Fluoro-3-[4-(2-hydroxy-ethanesulfonyl)-benzyl]-4-oxo-1-phenyl-1,4-dihydro-quinoline-2-carboxylic acid methyl ester. Worldwide Protein Data Bank; 2013. doi: 10.2210/pdb4g1w/pdb

[38] Goddard TD, Huang CC, Meng EC, Pettersen EF, Couch GS, Morris JH, et al. UCSF ChimeraX: Meeting modern challenges in visualization and analysis. Protein Sci. 2018;27(1):14–25. doi: 10.1002/pro.3235 28710774 PMC5734306

[39] Pettersen EF, Goddard TD, Huang CC, Meng EC, Couch GS, Croll TI, et al. UCSF ChimeraX: Structure visualization for researchers, educators, and developers. Protein Sci. 2021;30(1):70–82. doi: 10.1002/pro.3943 32881101 PMC7737788

[40] Meng EC, Goddard TD, Pettersen EF, Couch GS, Pearson ZJ, Morris JH, et al. UCSF ChimeraX: Tools for structure building and analysis. Protein Sci. 2023;32(11):e4792. doi: 10.1002/pro.4792 37774136 PMC10588335

[41] Viola A, Munari F, Sánchez-Rodríguez R, Scolaro T, Castegna A. The metabolic signature of macrophage responses. Front Immunol. 2019;10. doi: 10.3389/fimmu.2019.01462 PMC6618143 31333642

[42] Strelko CL, Lu W, Dufort FJ, Seyfried TN, Chiles TC, Rabinowitz JD, et al. Itaconic acid is a mammalian metabolite induced during macrophage activation. J Am Chem Soc. 2011;133(41):16386–9. doi: 10.1021/ja2070889 21919507 PMC3216473

[43] Marrocco A, Ortiz LA. Role of metabolic reprogramming in pro-inflammatory cytokine secretion from LPS or silica-activated macrophages. Front Immunol. 2022;13:936167. doi: 10.3389/fimmu.2022.936167 36341426 PMC9633986

[44] Tirichen H, Yaigoub H, Xu W, Wu C, Li R, Li Y. Mitochondrial reactive oxygen species and their contribution in chronic kidney disease progression through oxidative stress. Front Physiol. 2021;12:627837. doi: 10.3389/fphys.2021.627837 33967820 PMC8103168

[45] Yang W, Wang Y, Huang Y, Wang T, Li C, Zhang P, et al. Immune Response Gene-1 [IRG1]/itaconate protect against multi-organ injury via inhibiting gasdermin D-mediated pyroptosis and inflammatory response. Inflammopharmacology. 2024;32(1):419–32. doi: 10.1007/s10787-023-01278-x 37470905

[46] Wu YT, Xu WT, Zheng L, Wang S, Wei J, Liu MY, et al. 4-octyl itaconate ameliorates alveolar macrophage pyroptosis against ARDS via rescuing mitochondrial dysfunction and suppressing the cGAS/STING pathway. Int Immunopharmacol. 2023;118:110104. doi: 10.1016/J.INTIMP.2023.110104 37004345

[47] Bai B, Yang Y, Wang Q, Li M, Tian C, Liu Y, et al. NLRP3 inflammasome in endothelial dysfunction. Cell Death Dis. 2020;11(9):776. doi: 10.1038/s41419-020-02985-x 32948742 PMC7501262

[48] Zhang X, Lu X, Yu L, Gu Y, Qu F. Downregulation of NLRP2 inhibits HUVEC viability by inhibiting the MAPK signaling pathway. Mol Med Rep. 2019;19(1):85–92. doi: 10.3892/mmr.2018.9625 30431084 PMC6297776

[49] Zheng Y, Chen Z, She C, Lin Y, Hong Y, Shi L, et al. Four-octyl itaconate activates Nrf2 cascade to protect osteoblasts from hydrogen peroxide-induced oxidative injury. Cell Death Dis. 2020;11(9):772. doi: 10.1038/s41419-020-02987-9 32943614 PMC7499214

[50] Mills EL, Ryan DG, Prag HA, Dikovskaya D, Menon D, Zaslona Z, et al. Itaconate is an anti-inflammatory metabolite that activates Nrf2 via alkylation of KEAP1. Nature. 2018;556(7699):113–7. doi: 10.1038/nature25986 29590092 PMC6047741

[51] Zhang Q, Bai X, Wang R, Zhao H, Wang L, Liu J, et al. 4-octyl Itaconate inhibits lipopolysaccharide (LPS)-induced osteoarthritis via activating Nrf2 signalling pathway. J Cell Mol Med. 2022;26:1515–29. doi: 10.1111/JCMM.17185 35068055 PMC8899168

[52] Wang L, He C. Nrf2-mediated anti-inflammatory polarization of macrophages as therapeutic targets for osteoarthritis. Front Immunol. 2022;13:967193. doi: 10.3389/fimmu.2022.967193 36032081 PMC9411667

[53] Liu H, Feng Y, Xu M, Yang J, Wang Z, Di G. Four-octyl itaconate activates Keap1-Nrf2 signaling to protect neuronal cells from hydrogen peroxide. Cell Commun Signal. 2018;16(1):81. doi: 10.1186/s12964-018-0294-2 30442144 PMC6238317

[54] Tang C, Tan S, Zhang Y, Dong L, Xu Y. Activation of Keap1-Nrf2 signaling by 4-octyl itaconate protects human umbilical vein endothelial cells from high glucose. Biochem Biophys Res Commun. 2019;508(3):921–7. doi: 10.1016/j.bbrc.2018.12.032 30545629

[55] Xiang X, Chen J, Jiang T, Yan C, Kang Y, Zhang M, et al. Milk-derived exosomes carrying siRNA-KEAP1 promote diabetic wound healing by improving oxidative stress. Drug Deliv Transl Res. 2023;13(9):2286–96. doi: 10.1007/s13346-023-01306-x 36749479 PMC9904251

[56] Xiao W, Oldham WM, Priolo C, Pandey AK, Loscalzo J. Immunometabolic endothelial phenotypes: integrating inflammation and glucose metabolism. Circ Res. 2021;129(1):9–29. doi: 10.1161/CIRCRESAHA.120.318805 33890812 PMC8221540

[57] Gifre-Renom L, Daems M, Luttun A, Jones EAV. Organ-specific endothelial cell differentiation and impact of microenvironmental cues on endothelial heterogeneity. Int J Mol Sci. 2022;23(3):1477. doi: 10.3390/ijms23031477 35163400 PMC8836165

[58] Gobelli D, Serrano-Lorenzo P, Esteban-Amo MJ, Serna J, Pérez-García MT, Orduña A, et al. The mitochondrial succinate dehydrogenase complex controls the STAT3-IL-10 pathway in inflammatory macrophages. iScience. 2023;26(8):107473. doi: 10.1016/j.isci.2023.107473 37575201 PMC10416071

[59] Li W, Cai Z, Schindler F, Bahiraii S, Brenner M, Heiss EH, et al. Norbergenin prevents LPS-induced inflammatory responses in macrophages through inhibiting NFκB, MAPK and STAT3 activation and blocking metabolic reprogramming. Front Immunol. 2023;14:1117638. doi: 10.3389/fimmu.2023.1117638 37251401 PMC10213229

[60] Lian N, Mao X, Su Y, Wang Y, Wang Y, Wang Y, et al. Hydrogen-rich medium ameliorates lipopolysaccharides-induced mitochondrial fission and dysfunction in human umbilical vein endothelial cells (HUVECs) via up-regulating HO-1 expression. Int Immunopharmacol. 2022;110:108936. doi: 10.1016/j.intimp.2022.108936 35738091

[61] Stępińska O, Dymkowska D, Mateuszuk Ł, Zabłocki K. Lipopolysaccharide affects energy metabolism and elevates nicotinamide N-methyltransferase level in human aortic endothelial cells (HAEC). Int J Biochem Cell Biol. 2022;151:106292. doi: 10.1016/j.biocel.2022.106292 36038127

[62] Nicese MN, Bijkerk R, Van Zonneveld AJ, Van den Berg BM, Rotmans JI. Sodium butyrate as key regulator of mitochondrial function and barrier integrity of human glomerular endothelial cells. Int J Mol Sci. 2023;24(17):13090. doi: 10.3390/ijms241713090 37685905 PMC10487840

[63] Wang H, Sun X, Lu Q, Zemskov EA, Yegambaram M, Wu X, et al. The mitochondrial redistribution of eNOS is involved in lipopolysaccharide induced inflammasome activation during acute lung injury. Redox Biol. 2021;41:101878. doi: 10.1016/j.redox.2021.101878 33578126 PMC7879038

[64] Cipriano A, Viviano M, Feoli A, Milite C, Sarno G, Castellano S, et al. NADPH oxidases: from molecular mechanisms to current inhibitors. J Med Chem. 2023;66(17):11632–55. doi: 10.1021/acs.jmedchem.3c00770 37650225 PMC10510401

[65] Li R, Ma Y, Wu H, Zhang X, Ding N, Li Z, et al. 4-Octyl itaconate alleviates endothelial cell inflammation and barrier dysfunction in LPS-induced sepsis via modulating TLR4/MAPK/NF-κB signaling : 4-Octyl itaconate alleviates endothelial dysfunction. Mol Med. 2025;31(1):240. doi: 10.1186/s10020-025-01160-2 40524158 PMC12168283

[66] Ding Q, Sun T, Su W, Jing X, Ye B, Su Y, et al. Bioinspired multifunctional black phosphorus hydrogel with antibacterial and antioxidant properties: a stepwise countermeasure for diabetic skin wound healing. Adv Healthc Mater. 2022;11(12):e2102791. doi: 10.1002/adhm.202102791 35182097

[67] Ding Q, Jing X, Yao S, Su W, Ye B, Qu Y, et al. Multifunctional hydrogel loaded with 4-octyl itaconate exerts antibacterial, antioxidant and angiogenic properties for diabetic wound repair. Biomater Adv. 2022;139:212979. doi: 10.1016/j.bioadv.2022.212979 35882135

