# Supplementary figures and images for: The KEAP1/NRF2 axis controls LPS-induced oxidative stress, inflammasome activation and caspase-1 activity in human endothelial cells
Source: PLoS One. 2026 Feb 4;21(2):e0339928. doi: 10.1371/journal.pone.0339928 (PMC12872016; doi:10.1371/journal.pone.0339928)

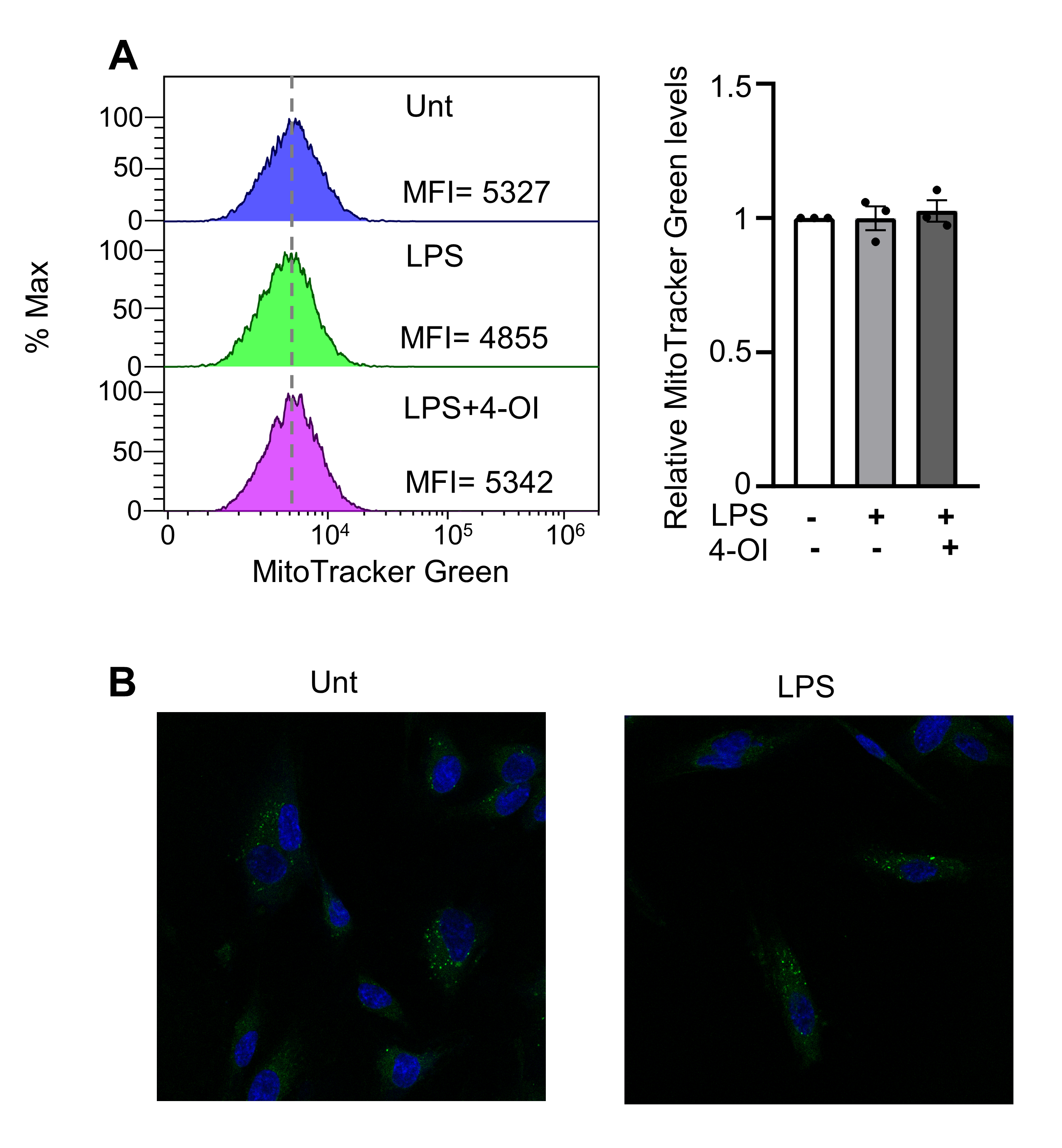

Supplement: S1 Fig — (A) When indicated, HUVECs were preincubated for 1 hour with 4-OI (50µM) and subsequently exposed to LPS (1 µg/ml) for 24 hours. Representative flow cytometry histogram of MitoTracker Green staining (left) and a graph showing relative MitoTracker Green levels (right). Each point represents a biological replicate. Data are shown as the mean ± SEM. (B) Representative immunofluorescence confocal images of untreated and LPS-treated HUVECs stained with MitoTracker Green (60 × magnification). (TIF) [file pone.0339928.s001.tif]

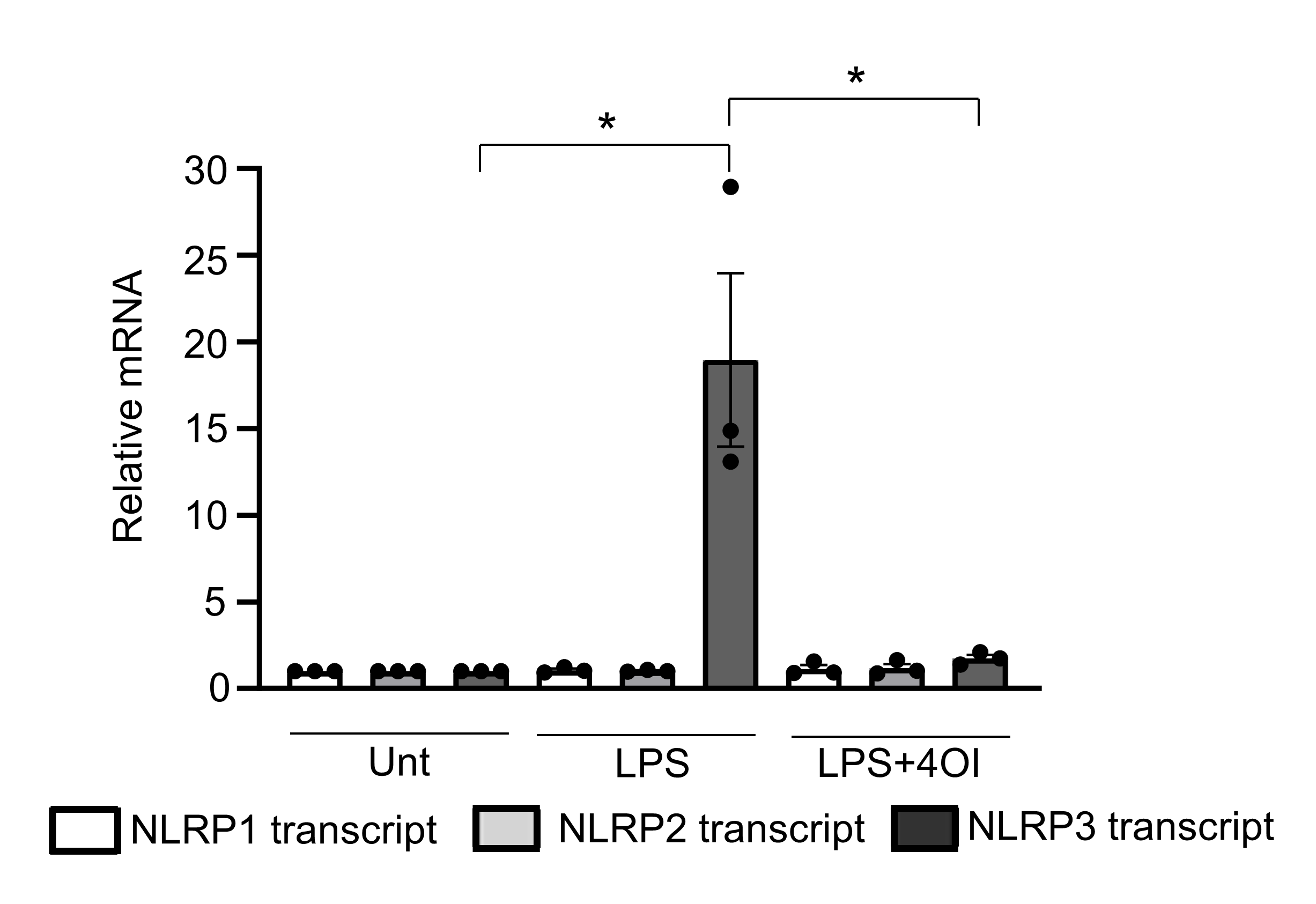

Supplement: S2 Fig — When indicated, HUVECs were pretreated with 4-OI (50 µM) for 1 hour prior to stimulation with LPS (1 µg/ml) for 6 hours. Bar graphs depict the relative expression levels of NLRP1, NLRP2 and NLRP3 transcripts relative to non-activated parental cells. Each point represents a biological replicate. Data are shown as the mean ± SEM. P values meaning: *, p < 0.05; **, p < 0.01; ***, p < 0.005; ****, p < 0.001. (TIF) [file pone.0339928.s002.tif]

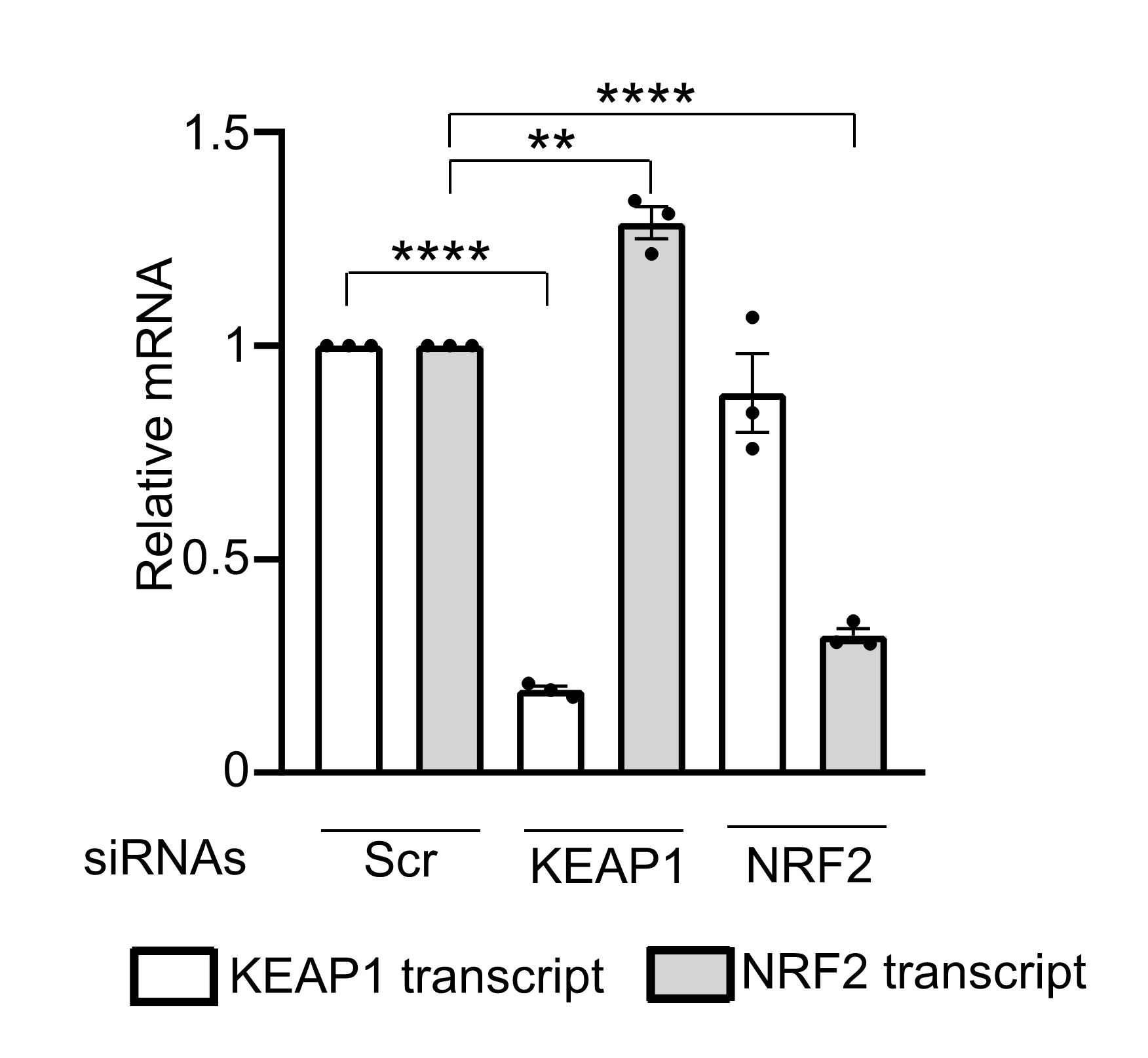

Supplement: S3 Fig — HUVECs were transfected with 30 nM scramble (Scr), KEAP1, or NRF2 siRNA, as indicated. Cells were collected at 48 h for transcript quantification by SYBR Green-based real-time PCR. Gene expression is expressed as fold change relative to cells treated with Scr siRNA. Each point represents a biological replicate. Data are shown as the mean ± SEM. P values meaning: *, p < 0.05; **, p < 0.01; ***, p < 0.005; ****, p < 0.001. (TIF) [file pone.0339928.s003.tif]

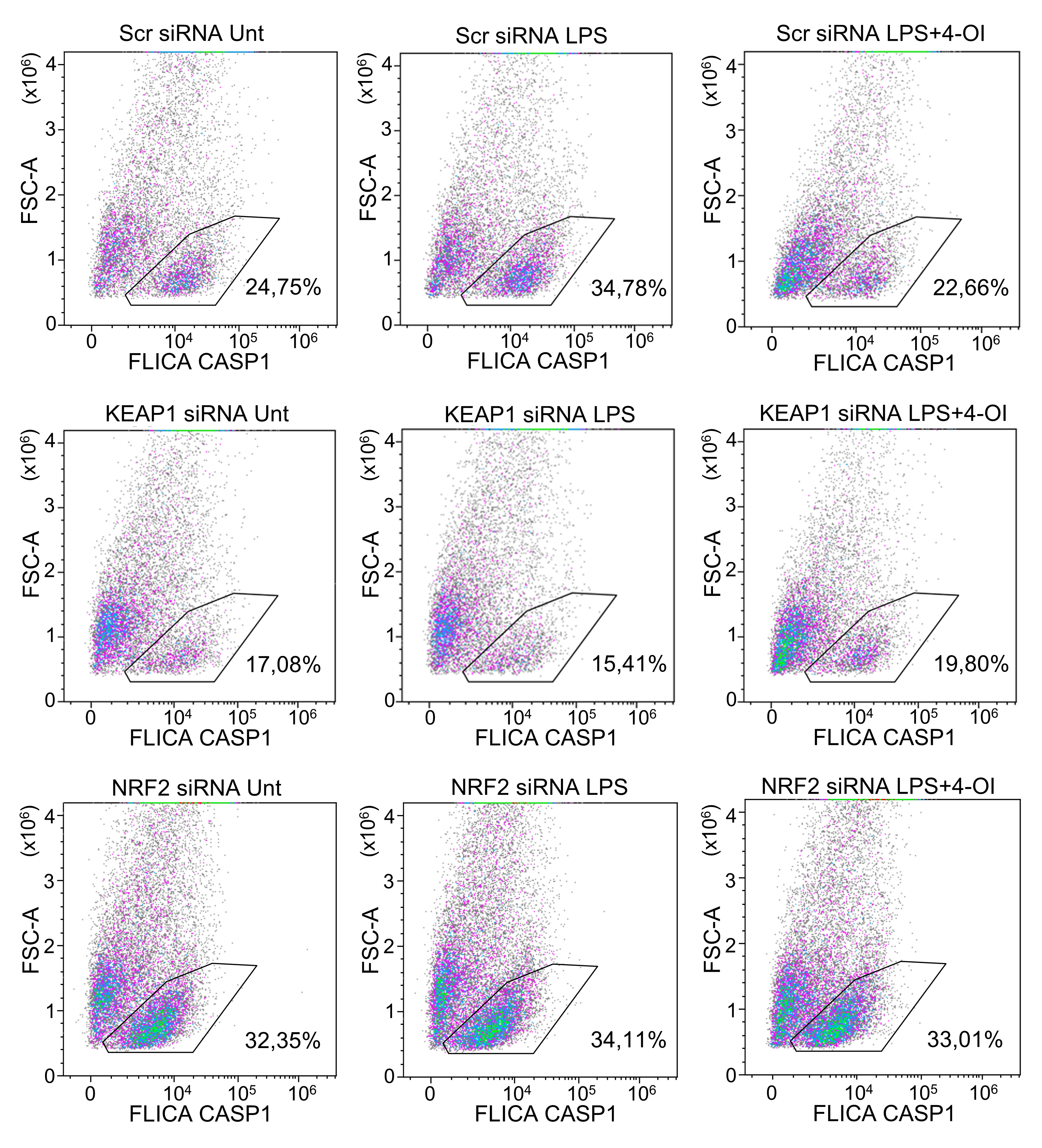

Supplement: S4 Fig — Following transfection with scramble (Scr), KEAP1, or NRF2 siRNAs, HUVECs were pretreated with 4-OI (50 µM) for 1 hour and subsequently exposed to LPS (1 µg/ml) for 24 hours, where indicated. Representative flow cytometry dot plots illustrating the gating strategy for caspase-1 positive cells (values are expressed in %). (TIF) [file pone.0339928.s004.tif]

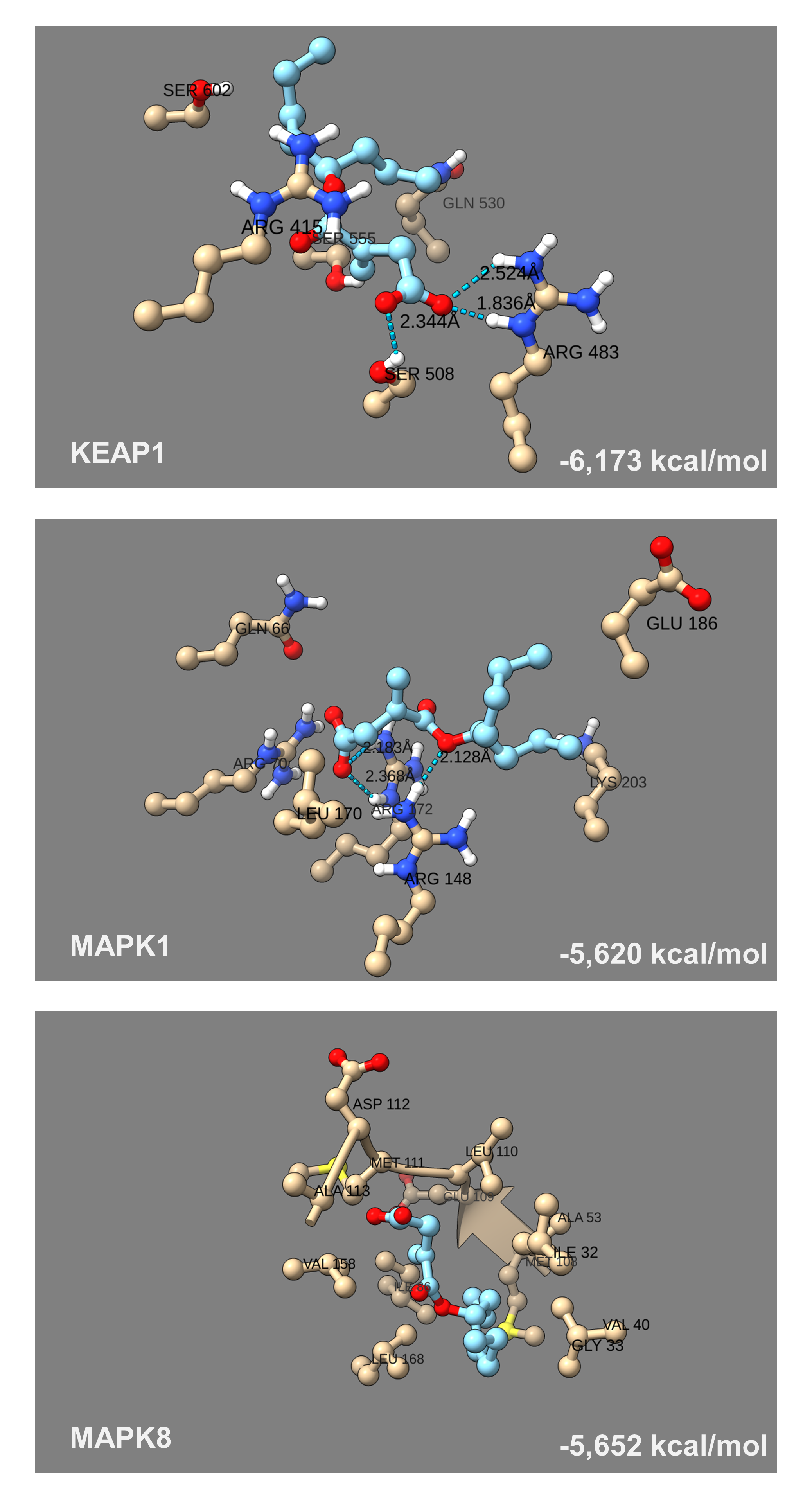

Supplement: S5 Fig — 3D model illustrating the interaction between the target proteins KEAP1, MAPK1, and MAPK8 and 4-OI (light blue). The binding affinities are shown in kcal/mol. Light-blue dashed lines indicate hydrogen-bond interactions; van der Waals forces are not explicitly depicted. (TIF) [file pone.0339928.s005.tif]

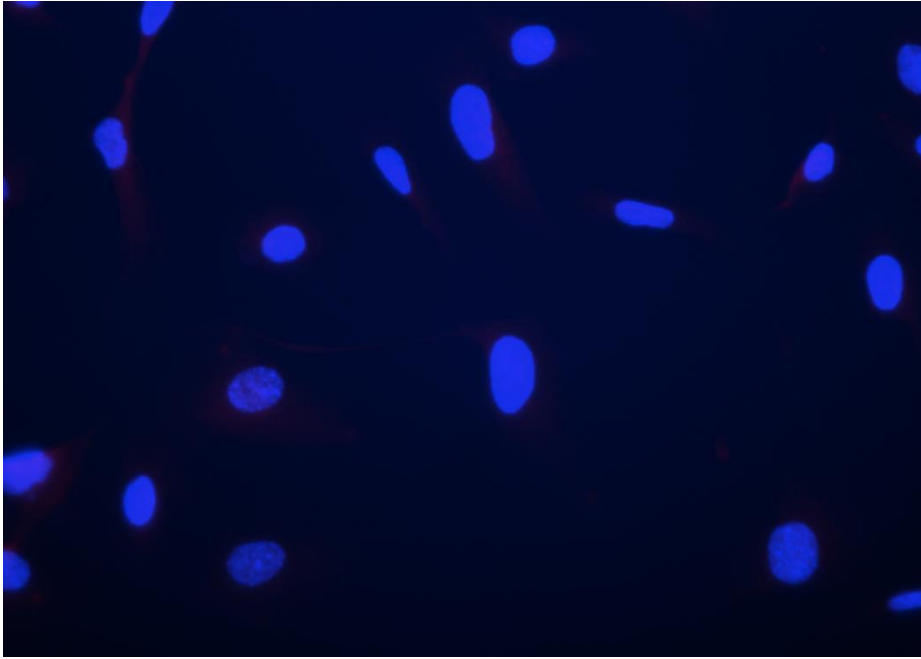

Fig 4A. Unt composite

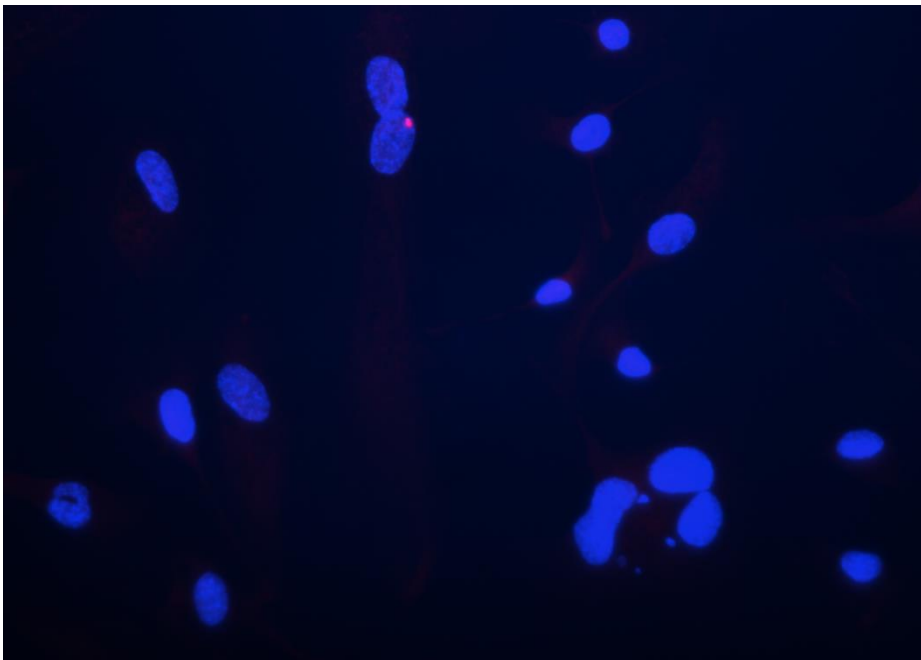

Fig 4A. LPS composite

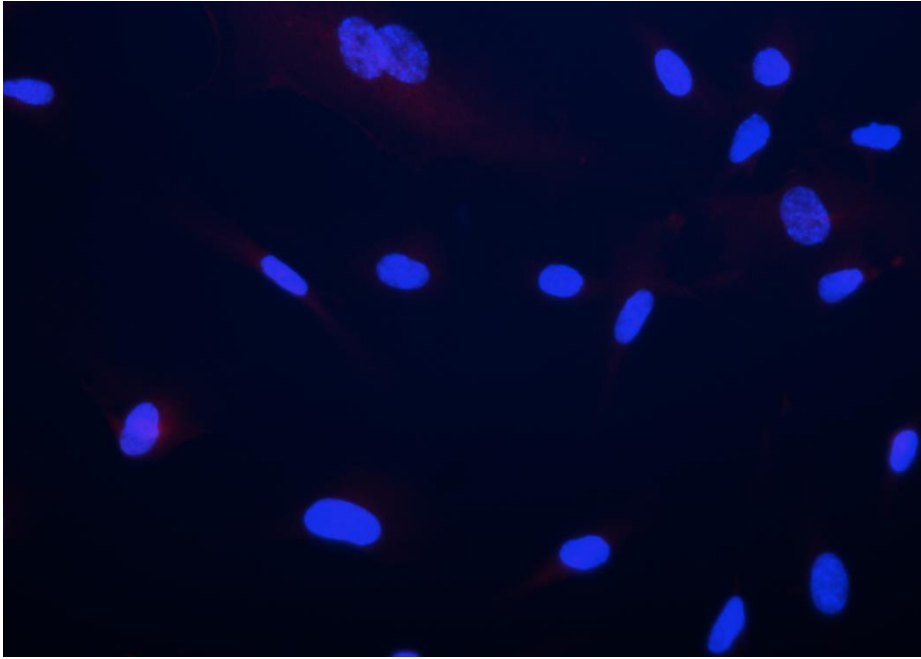

Fig 4A. LPS+4OI composite

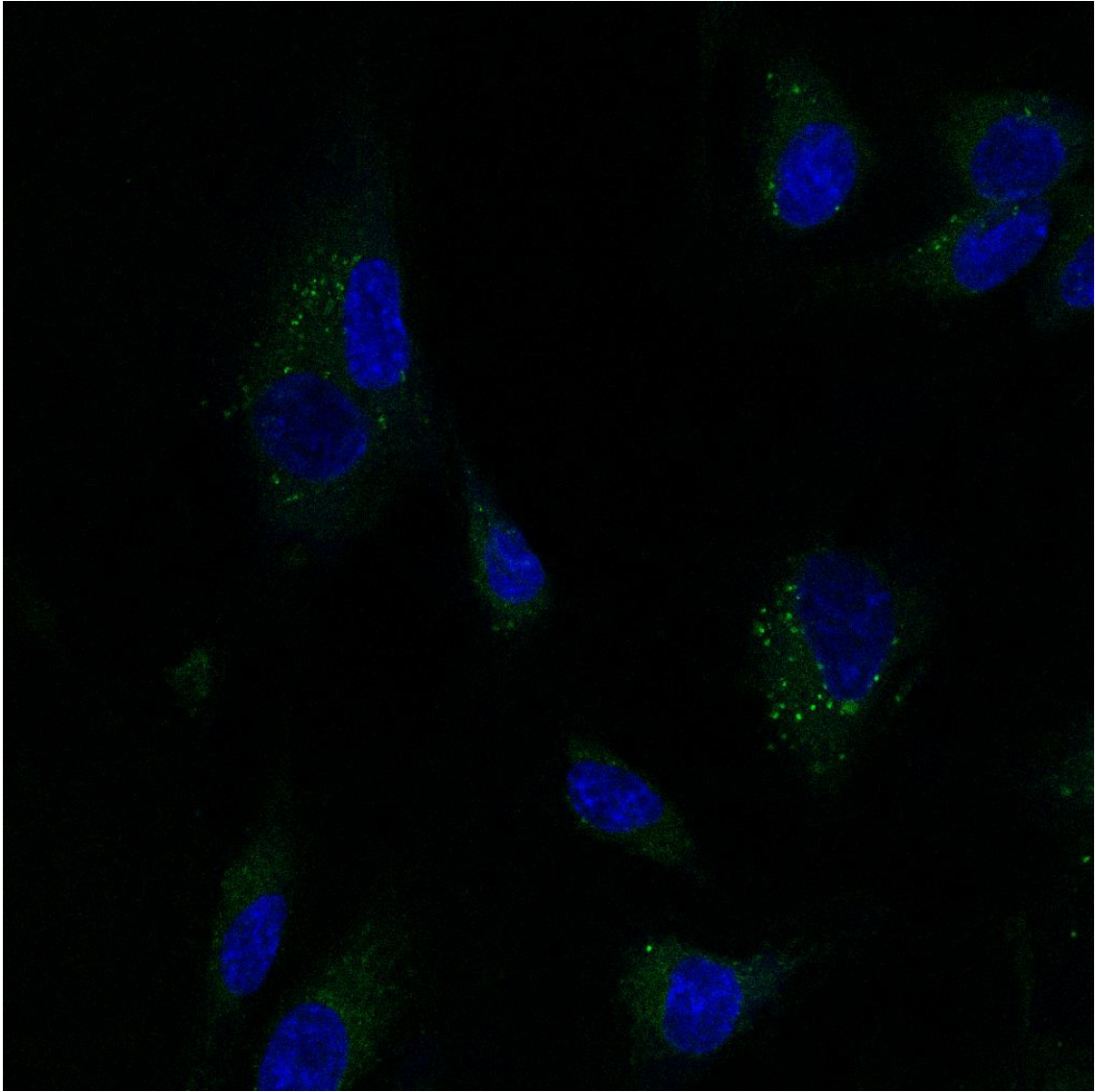

S1 Fig B Unt

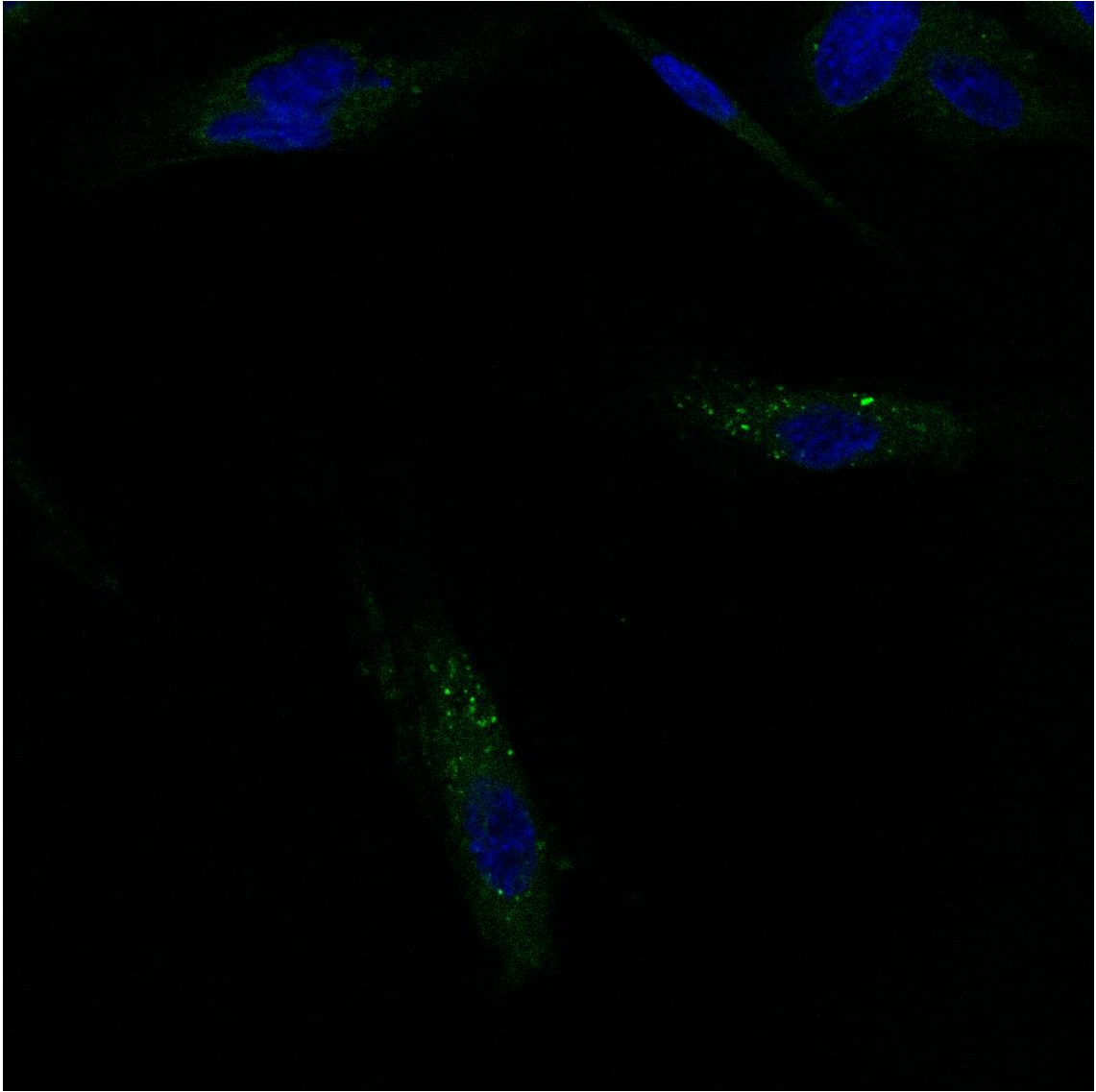

S1Fig B LPS

Supplement: S2 Dataset — (PDF) [file pone.0339928.s009.pdf]

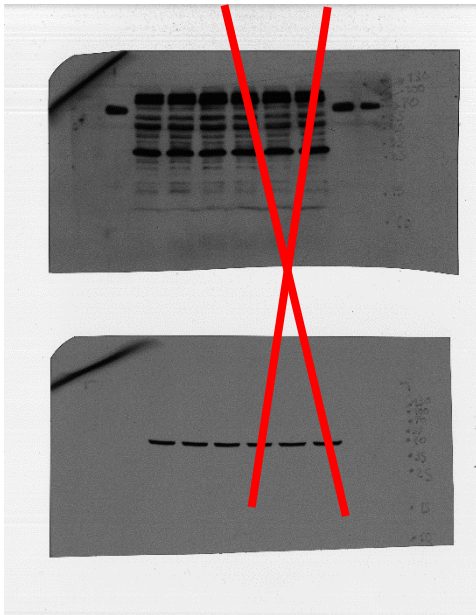

WB IL-1 $\beta$  and  $\beta$ -actin

Supplement: S3 Dataset — (PDF) [file pone.0339928.s010.pdf]
