# Supplementary material for: The KEAP1/NRF2 axis controls LPS-induced oxidative stress, inflammasome activation and caspase-1 activity in human endothelial cells
Source: PLoS One. 2026 Feb 4;21(2):e0339928. doi: 10.1371/journal.pone.0339928 (PMC12872016; doi:10.1371/journal.pone.0339928)
Supplement: S1 Table — (PDF) [file pone.0339928.s006.pdf]

Amplified gene	Primers
HPRT1	F: 5’-CCT GGC GTC GTG ATT AGT GAT GAT-3’
	R: 5’-AGC AAG ACG TTC AGT CCT GTC CAT-3’
KEAP1	F: 5’-ACC AGA GAC GTG GAC TTT CG-3’
	R: 5’-GTG TCT GTA TCT GGG TCG TAA C-3’
NRF2	F: 5’-TAG CCC CTG TTG ATT TAG ACG-3’
	R: 5’-TTC TGG ACT TGG AAC CAT GG-3’
HMOX1	F: 5’-TCA GGC AGA GGG TGA TAG AAG-3’
	R: 5’-TTG GTG TCA TGG GTC AGC-3’
NQO1	F: 5’-TTC CAG AAA GGA CAT CAC AGG-3’
	R: 5’-GGA ATA TCA CAA GGT CTG CGG-3’
GCLC	F: 5’-TTT TAC CGA GGC TAT GTG TCA G-3’
	R: 5’-GCT GTC TAT TGA GTC ATA TCG GG-3’
NLRP1	F: 5’-CCG CTG ACC CCA CTT TAT ATG-3’
	R: 5’-CAA CGT AGA ACT CCG AGA ACA G-3’
NLRP2	F: 5’-ACA AAT AGC CTC TGA CAC CTG-3’
	R: 5’-TCA TCC TGG TCA TTG CCT TG-3’
NLRP3	F: 5’-GTG TTT CGA ATC CCA CTG TG-3’
	R: 5’-TCT GCT TCT CAC GTA CTT TCT G-3’
