# Supplementary material for: The KEAP1/NRF2 axis controls LPS-induced oxidative stress, inflammasome activation and caspase-1 activity in human endothelial cells
Source: PLoS One. 2026 Feb 4;21(2):e0339928. doi: 10.1371/journal.pone.0339928 (PMC12872016; doi:10.1371/journal.pone.0339928)
Supplement: S2 Table — (PDF) [file pone.0339928.s007.pdf]

PROTEIN	CENTER_X	CENTER_Y	CENTER_Z	SIZE_X	SIZE_Y	SIZE_Z
KEAP1	15.668	49.744	38.392	60	74	70
MAPK1	18.912	-3.613	16.474	100	48	60
MAPK8	-3.513	5.407	-23.318	100	48	102
